# Transcriptomic Analysis of Induced Pluripotent Stem Cells Derived from Patients with Bipolar Disorder from an Old Order Amish Pedigree

**DOI:** 10.1371/journal.pone.0142693

**Published:** 2015-11-10

**Authors:** Kwi Hye Kim, Jiangang Liu, Rachelle J. Sells Galvin, Jeffrey L. Dage, Janice A. Egeland, Rosamund C. Smith, Kalpana M. Merchant, Steven M. Paul

**Affiliations:** 1 Lilly Research Laboratories, Eli Lilly and Company, Indianapolis, Indiana, United States of America; 2 Department of Psychiatry and Behavioral Sciences, University of Miami, Miller School of Medicine, Miami, Florida, United States of America; 3 Mind and Brain Institute, Weill Cornell Medical College, New York, New York, United States of America; Albert Einsten College of Medicine, UNITED STATES

## Abstract

Fibroblasts from patients with Type I bipolar disorder (BPD) and their unaffected siblings were obtained from an Old Order Amish pedigree with a high incidence of BPD and reprogrammed to induced pluripotent stem cells (iPSCs). Established iPSCs were subsequently differentiated into neuroprogenitors (NPs) and then to neurons. Transcriptomic microarray analysis was conducted on RNA samples from iPSCs, NPs and neurons matured in culture for either 2 weeks (termed early neurons, E) or 4 weeks (termed late neurons, L). Global RNA profiling indicated that BPD and control iPSCs differentiated into NPs and neurons at a similar rate, enabling studies of differentially expressed genes in neurons from controls and BPD cases. Significant disease-associated differences in gene expression were observed only in L neurons. Specifically, 328 genes were differentially expressed between BPD and control L neurons including GAD1, glutamate decarboxylase 1 (2.5 fold) and SCN4B, the voltage gated type IV sodium channel beta subunit (-14.6 fold). Quantitative RT-PCR confirmed the up-regulation of GAD1 in BPD compared to control L neurons. Gene Ontology, GeneGo and Ingenuity Pathway Analysis of differentially regulated genes in L neurons suggest that alterations in RNA biosynthesis and metabolism, protein trafficking as well as receptor signaling pathways may play an important role in the pathophysiology of BPD.

## Introduction

Bipolar disorder (BPD) is a highly heritable mood disorder characterized in many patients by dramatic and unpredictable mood swings between depression and mania or hypomania. Nearly 50% of bipolar patients attempt suicide and half of them succeed [1]. Available therapeutic agents effectively treat symptoms in only a subset of patients, accounting for the high morbidity and mortality associated with BPD [2,3]. Recent research efforts have focused on identifying the underlying genes and genetic factors for BPD with the goal of using these molecular genetic insights to design therapeutics with greater efficacy [4,5].

Studies on genetic or population isolates offer a powerful approach to identifying genetic variants accounting for disease heritability due to their relatively reduced genetic, environmental and phenotypic variability. The Old Order Amish of Lancaster County represent a genetic isolate of European ancestry where a number of disease causing genetic mutations have been identified including several Mendelian disorders [6,7]. We have recently reported an in-depth genetic analysis of a large, multigenerational Amish pedigree with BPD [8]. Although the small sample size may have hindered the identification of risk variants or a common etiologic pathway, a set of credible candidate genes were identified for further examination in other Amish pedigrees or in large-scale population-based studies. In addition to genetic studies, gene expression studies on postmortem brain tissue from BPD patients and controls have been conducted as an orthogonal approach to elucidate mechanisms underlying BPD [9–15]. A major caveat of post mortem brain studies, however, is the difficulty in differentiating disease etiology-associated changes (e.g. gene expression) from those caused by post-mortem artifact, life-long illness or prior drug treatment. Recent advances in human induced pluripotent stem cell (iPSC) technology, on the other hand, have the potential to address some of the shortcomings of postmortem studies. For example, iPSC-derived neurons from neurodegenerative diseases with Mendelian inheritance such as familial Amyotrophic Lateral Sclerosis (ALS) and Parkinson’s disease recapitulate key pathological mechanisms associated with the disorders [16–18]. Interestingly, even in a complex psychiatric disorder such as schizophrenia, iPSC-derived neurons have been shown to display morphological and gene expression changes that may be relevant to underlying disease biology [19,20].

Recent studies have applied iPSC technology to the study of BPD. Chen et al [21] generated iPSCs from 3 unrelated patients with Type 1 BPD and saw differential gene regulation in neurons derived from these cell lines when compared to neurons from lines derived from unaffected, unrelated controls. Madison et al [22] generated iPSCs from 2 affected and 2 unaffected individuals in a pedigree enriched for BPD. Comparison of gene expression profiles between BPD and control NPs and BPD and control neurons identified differentially regulated genes. To gain further insight into BPD, we chose to generate iPSCs and their derivatives from individuals within a 5 generation Old Order Amish pedigree with high prevalence of BPD [8]. We reasoned that transcriptomic analysis of iPSC-derived neurons from affected and unaffected first-degree relatives might not only offer a better approach to gain insights into the molecular changes of BPD by reducing both genetic and non- genetic variability, but moreover, might leverage the existing genetic data from this pedigree. The iPSC lines from 4 affected and 4 unaffected relatives were differentiated into neuroprogenitors (NPs) and neurons *in vitro*. Using microarray analysis, we confirmed stages of differentiation of the iPSCs to neurons by characteristic gene expression patterns, and observed a subset of differentially expressed genes (DEGs) between BPD subjects and their healthy siblings only in L neurons. Hierarchical clustering and pathway analyses revealed differences in genes involved in RNA biosynthesis, protein trafficking and receptor signaling pathways.

## Methods

### Diagnostic Guidelines and Reliability

When the Amish Study, which includes the cases reported here, began in 1976 it established a project Psychiatric Board of five clinicians charged with diagnosis of patient case materials using strict research diagnostic criteria (both RDC and DSM III–IV) blind to existing medical “treatment opinions” of practitioners and unaware of family relatedness. Each BPD case was re-evaluated blindly at least twice over the decades resulting in consensus diagnoses. Follow-up of BPD patients and their unaffected relatives for genetic research, including those used for the present study, was carried out yearly and has spanned 2–3 decades [23]. A second diagnostic procedure tracked course-of-illness by both the Board and a project Seasonality Panel comprised of three psychiatrists, who coded episodes that extended beyond 20–30 years for the patients in this study. Medical records documented the extent to which lithium was an effective mood stabilizing medication for the BPD patients used to generate iPSC lines. Data for 87 individuals from four large Amish families, the largest being core Pedigree 110, location of the present research subjects, has been described [24]. The project Board and Panel were in agreement regarding medication compliance and the effectiveness of lithium. Several independent sources provided evidence about breakthrough episodes (due to poor compliance) and subsequent recovery with lithium as the mood stabilizing drug.

### Patient Population

**Fig 1:**
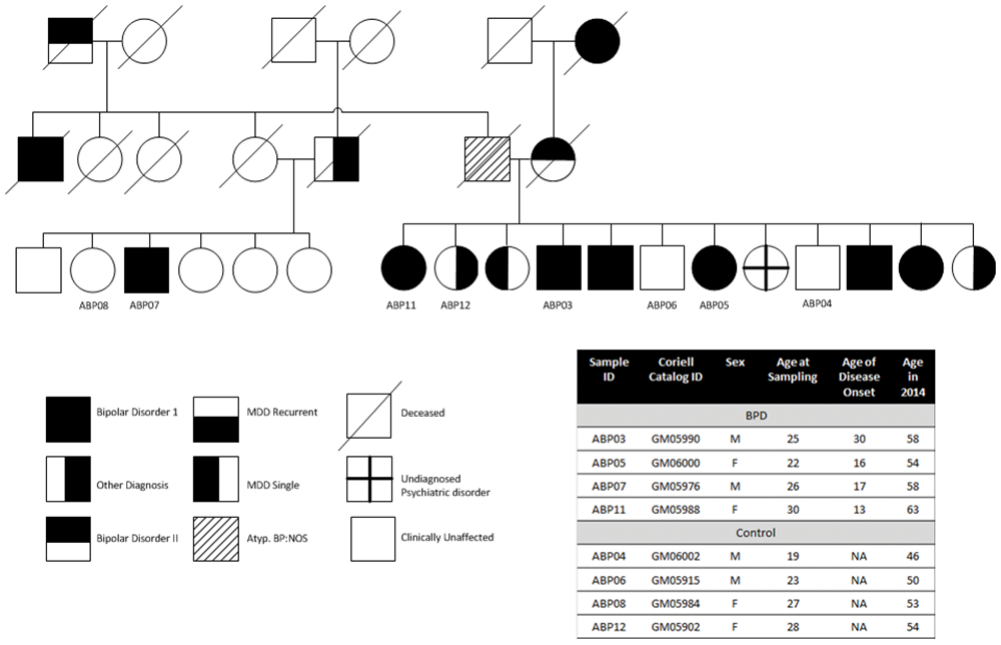
Selection of fibroblasts from the NIGMH repository for generation of iPSCs. Fibroblasts from patients diagnosed with Type I BPD and their sibling controls of Old Order Amish pedigree were obtained from the NIGMS Human Genetic Cell Repository at Coriell Institute for Medical Research for reprogramming into iPSCs using the Sendai virus method. The pedigree has been updated according to DSM-IV. Circles and squares represent females and males, respectively. MDD = Major Depressive Disorder; Atyp. BP:NOS = Atypical Bipolar (not otherwise specified). Other diagnosis for the patient from which ABP12 was derived was post-partum depression.

A multi-generational Old Order Amish pedigree with high prevalence of Type I BPD was chosen for the current study [7,25,26]. Fig 1 shows the pedigree and associated demographics. Fibroblast cell lines derived from four BPD patients (ABP03, 05, 07 and 11) and four unaffected siblings (ABP04, 06, 08 and 12), were obtained from the NIGMS Human Genetic Cell Repository at Coriell Institute for Medical Research (all samples in this biorepository have fulfilled the required informed consent guidelines detailed at https://catalog.coriell.org/1/NIGMS/About/Submitting-Samples). The 4 BPD patients all received high rating for classic presentation of the disease as determined by special coding of the project Board. Their ages at onset of BPD (Fig 1) are reflective of the range for onset age in the total BPD Amish sample. The unaffected control samples had a similar age range. All controls were well beyond the age of risk for BPD in these families.

#### Generation of human iPSCs

Fibroblasts (passages 3–8) were reprogrammed using CytoTune^®^-iPS kit (Life Technologies) following methods described previously [27]. Three iPSC lines with characteristic morphology were generated for each patient fibroblast cell line. Cytogenetic analysis (Cell Line Genetics) on 20 G-banded metaphase cells was performed from each cell line and all 20 cells demonstrated normal karyotype. No abnormal cells were detected. We randomly chose one clone from each patient for subsequent studies.

Selected iPSCs were expanded and cultured on irradiated mouse embryonic fibroblasts (MEF) (GlobalStem) in ES media, which contains DMEM/F12 (1:1) (HyClone) with 20% Knockout serum replacement (Invitrogen), 1X penicillin/Streptomycin (10U/ml/10μg/ml, Hyclone), 1X GlutaMax (Invitrogen), 1X Non-essential amino acids (Hyclone), 55 μM 2-mercaptoethanol (Gibco) and 10ng/ml human bFGF (R&D Systems) with daily media replacement.

### Differentiation of iPSC into neurons

Before initial differentiation, iPSCs were transferred to plates coated with Matrigel^™^ (BD Bioscience) following the manufacturer’s instruction and maintained in mTeSR1 media (StemCell Technologies) until MEF feeder cells were eliminated. Neural Induction Media (NIM) was prepared to contain DMEM/F12 (1:1) with 1X N2 supplements (Invitrogen), 1X penicillin/Streptomycin (10U/ml/10μg/ml, Hyclone), 1X GlutaMax (Invitrogen), 1X Non-essential amino acids (Hyclone). Neural Differentiation Media (NDM) was prepared by adding 1X N2 supplements, 1X B27 without vitamin A (Invitrogen), 1X penicillin/Streptomycin, 1X GlutaMax, 1X Non-essential amino acids (Hyclone) to Neurobasal media (Life Technologies).

Human iPSCs were differentiated using published protocols [28,29] with few modifications to increase differentiation efficiency as illustrated in S1 Fig. Briefly, hiPSCs at between 10 and 25 passages were rinsed with 1X Ca^2+^/Mg^2+^ free Dulbecco’s Phosphate-Buffered Saline (HyClone; DPBS) and then treated with Accutase (MP Biomedicals) for 5 ~ 7 minutes at 37°C to make a single cell suspension. Live cells were counted using Vi-Cell (Beckman-Coulter). At day 0 of differentiation, three million cells were added to each well of an AggreWell800 plate (Stem Cell Technologies, Inc.) following manufacturer’s protocol in NIM with 250 ng/ml noggin (R&D Systems) and 10 μM SB431542 (R&D Systems). 50 ~ 75% of the media was replaced daily for 5 days with fresh NIM supplemented with noggin and SB431532. On the fifth day of differentiation, cell clusters were collected from a well of the AggreWell800 plate and re-plated in 6-well plates coated with Matrigel^™^ in NIM supplemented with noggin and SB431532. Media was replaced every other day for an additional four to five days until neural rosettes composed of NPs were detected in most areas of the wells.

NPs were dissociated from the neural rosettes with Accutase and expanded by culturing on Matrigel-coated plates at >0.1 x 10^6^ cells/cm^2^ or seeded at 0.33 x 10^6^ cells/ml in Ultra-Low Attachment plates (Corning) for suspension culture in NDM supplemented with 10 ng/ml hLIF (Millipore) and 20 ng/ml bFGF (R&D Systems). After two passages, NPs were dissociated as single-cell suspensions and cryopreserved for future use in 50% NDM, 40% Knockout serum replacement and 10% dimethyl sulfoxide (Sigma).

To further differentiate NPs into neurons, NPs were cultured on plates coated with 0.1% poly-l-ornithine (Sigma) and 10ng/ml of laminin (Invitrogen) in NDM without hLIF and bFGF at 25,000 ~ 50,000 cells/cm^2^. Media was replaced every 2 ~ 3 days. Differentiation was continued for up to 4 weeks in culture. Cells were harvested and RNA samples were obtained after two weeks to represent an early time point of neuronal differentiation (E) and four weeks to represent a later time point (L) of neuronal differentiation.

### Immunocytochemistry

Immunocytological analysis of iPSCs and embryoid bodies was as described previously [30]. Neural rosettes, NPs and neurons were fixed with 4% paraformaldehyde and stained with Pax6 (Covance), Nestin (Covance), MAP2 (AbCam) and β-tubulin III (TUJ1 clone; Covance) antibodies using standard methods.

### RNA isolation for gene expression studies

Undifferentiated iPSCs, iPSC-derived NPs or neurons at E and L stage of differentiation were rinsed with 1X DPBS followed by RNA extraction using RNeasy Mini kit (Qiagen) using the manufacturer’s protocol. RNA was quantitated using a Nanodrop Spectrometer ND-1000 (Thermo Scientific).

### Reverse transcription and quantitative reverse transcription PCR

Reverse transcription PCR (RT-PCR) analyses for iPSC and embryoid body (EB) characterization were as described previously [30,31], using the same primers, except for PAX6, FLK1 and GATA2 which are listed in Part A of S1 Table. For all quantitative reverse transcription PCR (qRT-PCR) analyses, complementary DNA (cDNA) synthesis was performed using High Capacity cDNA reverse transcriptase kit (Life Technologies), according to the manufacturer’s instructions. Quantitative PCR analysis of reverse transcribed cDNA was conducted in triplicate using the Applied Biosystems ABI 7900 HT system with TaqMan^®^ Assays and TaqMan^®^ Gene Expression Master Mix (Life Technologies, Carlsbad, CA) with primer probes as listed in Part B of S1 Table.

### Microarray analysis

Samples for messenger RNA (mRNA) profiling studies were processed by Asuragen, Inc. (Austin, TX, USA) using GeneGhip^®^ Human Genome U133 (Affymetrix, Santa Clara, CA) according to the company's standard operating procedures as described previously in detail [32].

### Statistical and bioinformatics data analysis

A summary of the image signal data, detection calls and gene annotations for every gene interrogated on the arrays was generated using the Affymetrix Statistical Algorithm MAS 5.0 (GCOS v1.3) (scaling factor = 1500) and quantile normalization across samples was applied. Log2 transformation and mean-centered standardization was further performed. For genes with multiple probes on the chip, signal intensities were averaged. Each probe ID was converted and collapsed into gene symbols (http://www.genenames.org/). The final 15391 genes that were reliably detected on at least 80% of the arrays with a signal intensity of 64 or greater were used for further analysis.

For Principal Component analysis (PCA), the replicates per group and time points were aggregated by calculating their means and the first two principal components (PCs) were used to visualize the global similarity of the data. Analysis of variance (ANOVA) was applied to determine differentially expressed genes (DEGs) in pair-wise comparisons of the groups (e.g., BPD vs. control for NP, E and L, respectively) and combined for all groups. The ANOVA assessed the main effects of disease status (BPD vs. control) and differentiation stages (NP, E, L) as well as interactions between the two variables. ANOVA was conducted for p-values for each of the three effects (disease status, differentiation stage, and interaction between two variables) along with adjusted p-values for multiple testing using Benjamini-Hochberg-false discovery rate (FDR) correction. Differentially Expressed Genes (DEG) were defined as the probes which had FDR < = 0.2, mean signals > = 250 in at least one group (control or BPD) and absolute fold change > = 1.5 in all chips.

#### Hierarchical Clustering

The gene sets constituting pathways relevant to neuronal development and differentiation were retrieved from Molecular Signatures Database (MsigDB 3.0, C2 & C5) [33]. Each pathway-specific data set was analyzed by hierarchical average-linkage clustering. The clustering was performed using Gene Cluster 3.0 (http://bonsai.ims.utokyo.ac.jp/~mdehoon/software/cluster/) or using R programs.

The resulting numerical output was used in R package (“gplots”) to generate the associated heat maps and clustering dendrograms.

#### Enrichment analysis

Enrichment analyses of Gene Ontology (GO), GeneGo, KEGG and Ingenuity Pathway Analysis (IPA) pathways were performed for DEGs using the hypergeometric test and Fisher’s Exact Test. To account for multiple hypothesis testing, FDR correction (FDR = 5%), was applied according to Benjamini-Hochberg.

## Results

### Characterization of iPSCs

Cytogenetic analysis confirmed that all 8 iPSC lines were karotypically normal. Gene expression of pluripotency markers and inactivation of Sendai-virus particles were confirmed in all iPSC lines by qRT-PCR as described by Park et al. [30] and Chan et al [31] (Panel A of S2 Fig). Protein expression of pluripotency markers was demonstrated by immunocytochemistry (Panel B of S2 Fig). To confirm the capacity of iPS cells to spontaneously differentiate into three germ layers, EBs were generated and maintained in suspension for spontaneous differentiation. Expression of genes specific for three germ layers was demonstrated by RT-PCR (Panel C of S2 Fig).

### Differentiation of iPSCs to NPs and neurons

**Fig 2:**
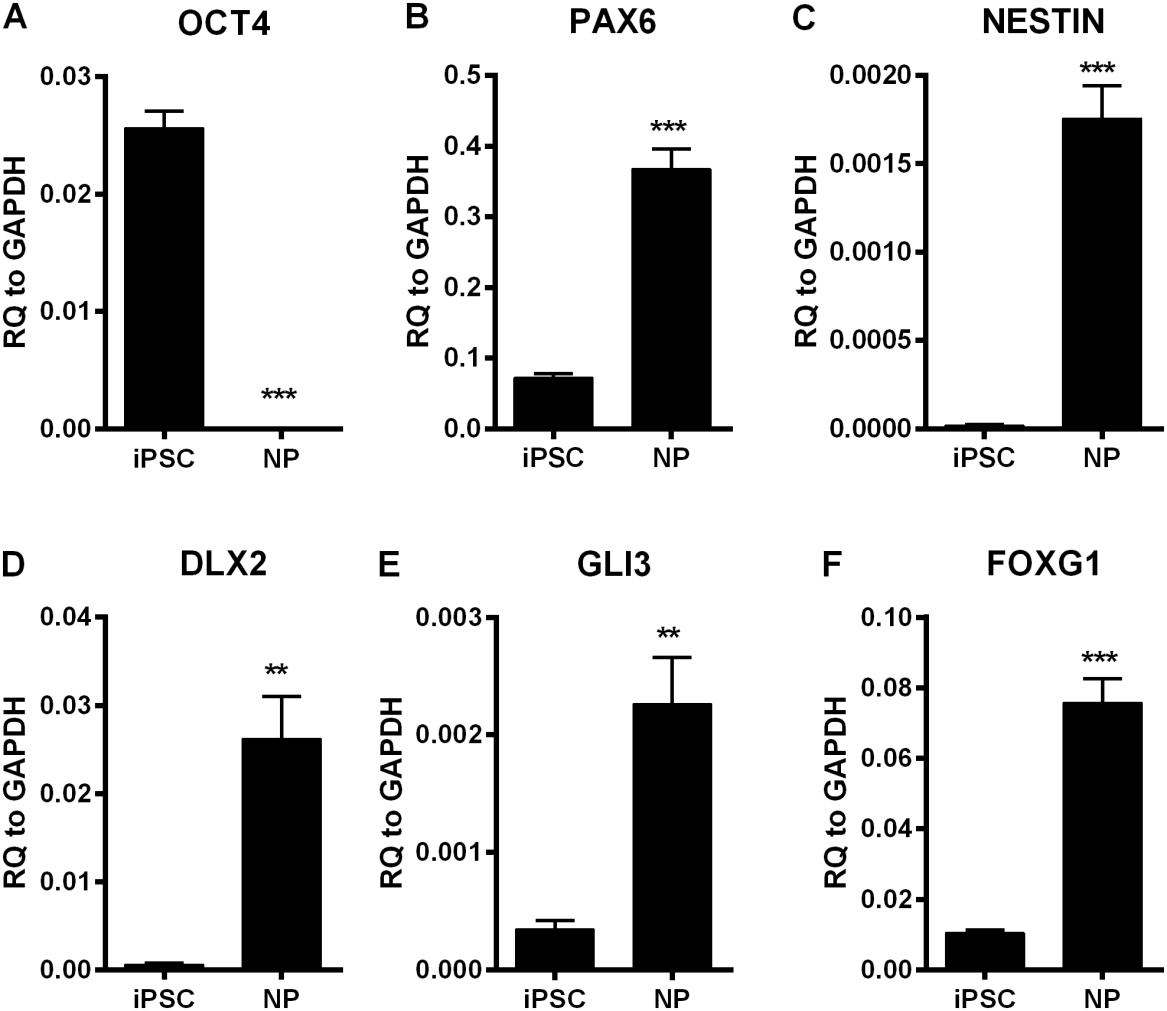
Quantitative RT-PCR of RNA samples from iPSCs and NPs. RNA samples were collected from iPSC and NPs and gene expression of markers of developmental stages were evaluated. Changes in pluripotency and neuroprogenitor markers were measured (A-F). Each bar represents mean ± standard error, n = 8. Statistical analysis was performed using unpaired *student t-test*. *** p < 0.0001, ** p < 0.001.

Four BPD patient-derived iPSC clones (ABP03, 05, 07 and 11) and four unaffected sibling-derived (control) iPSC clones (ABP04, 06, 08 and 12), were differentiated into neuronal progenitors (NPs) and neurons as described in Materials and Methods section. S1 Fig shows the key characteristics of the cultures along the stages of neuronal differentiation. L neuronal cultures exhibited characteristic neuronal morphology including axons, dendrites and neurites in addition to positive immunostaining for MAP2 and β-tubulin III (S1 Fig). For microarray studies and further characterization, RNA samples from the four BPD and four controls were collected from NP, E neurons (2 weeks of differentiation) and L neurons (4 weeks of differentiation) as well as undifferentiated iPSCs. Expression of markers of pluripotency and neuronal differentiation were examined using qRT-PCR. The differentiation of iPSCs into NPs resulted in a dramatic decrease in expression of the pluripotency marker, OCT4 (Fig 2A). At the same time, PAX6 and NESTIN expression, early markers of neuroectoderm, increased indicating desired lineage specification (Fig 2B and 2C)[34]. The generation of NPs was further confirmed by increased expression of the dorsoventral neuronal specification markers DLX2, GLI3 and FOXG1 (Fig 2D–2F) [35,36]. Gene expression for endodermal (AFP and SOX17) [37] and mesodermal (T/Brachyury) markers [38] decreased during differentiation (data not shown), consistent with differentiation towards the neuroectodermal lineage.

**Fig 3:**
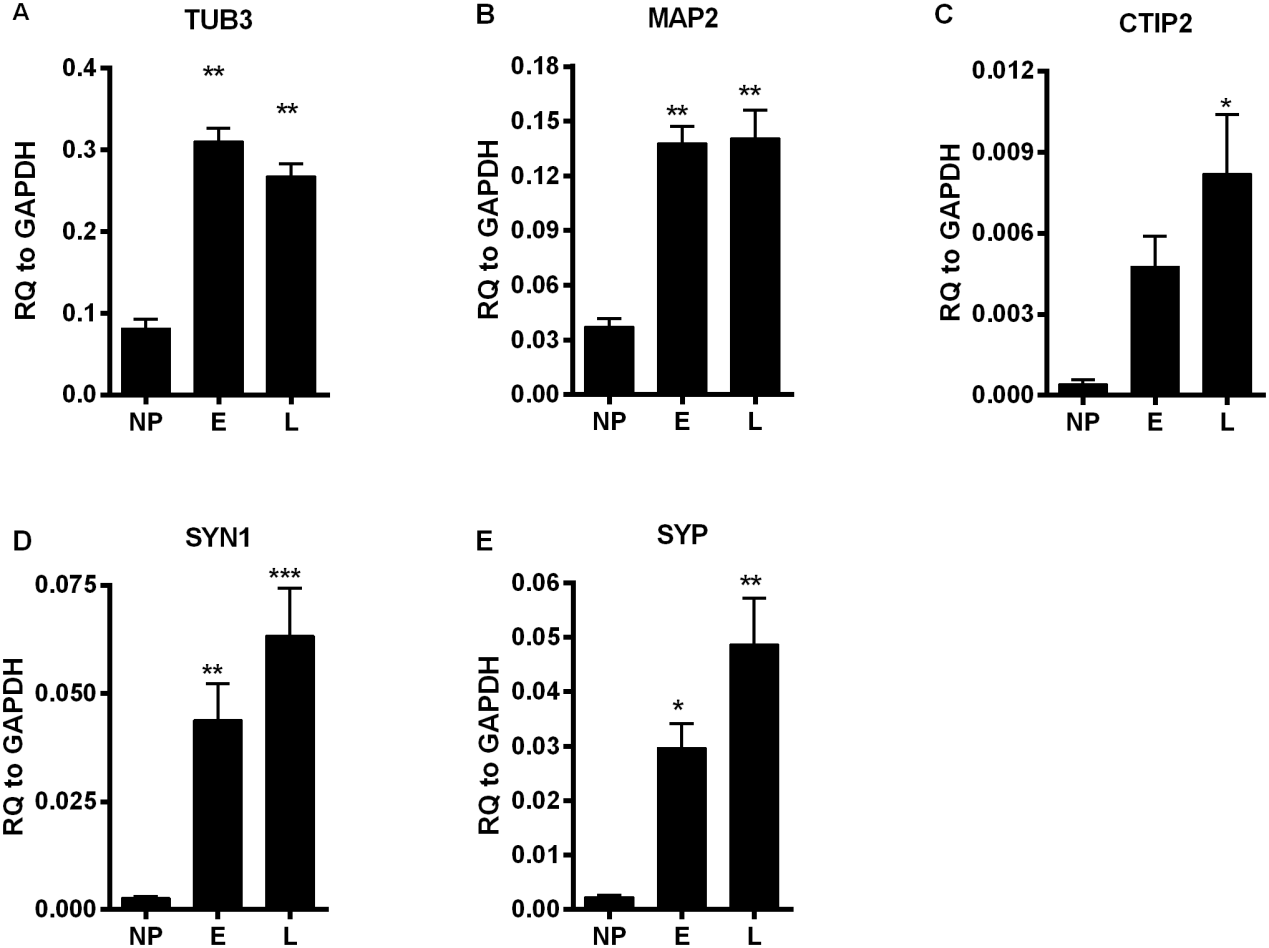
Quantitative RT-PCR of RNA samples from NPs and neurons. RNA samples were collected from NPs, E and L neurons and markers of neuronal gene expression were evaluated. Pan-neuronal markers, (A,B), cortical neuronal marker (C), and synaptic markers (D,E) were evaluated. Each bar represents mean ± standard error (n = 8, combining 4 BPD and 4 controls). One-way ANOVA was performed and Bonferroni’s Multiple Comparison post-hoc test was done. * p < 0.01, ** p < 0.001, *** p < 0.0001 compared to NP.

Neuronal differentiation was initiated by growth factor withdrawal from NPs. Successful neuronal commitment was demonstrated in E neurons by an increase in the expression of pan-neuronal markers, tubulin β-III (TUB3) and microtubule associated protein 2 (MAP2) [39,40] (Fig 3A and 3B). Expression of CTIP2, a marker for deep cortical neurons [41], was significantly increased by four weeks of neuronal differentiation (L) (Fig 3C). Expression levels of the synaptic markers, synapsin 1 (SYN1) and synaptophysin (SYP), was positively correlated with the time of neuronal differentiation. (Fig 3D and 3E). Gene expression of several neuronal ion channel proteins also tended to increase as NPs matured into neurons (S3 Fig), although some of the markers did not reach statistical significance. Collectively, the gene expression analyses indicate that E neurons already exhibit key neuronal identifiersbut markers for neuronal function continue to increase their expression level with further differentiation to L neurons.

### Differentially Regulated Genes (DEGs) in Neuronal Differentiation

RNA samples isolated from NP, E and L neurons of the four BPD and four control donors were subjected to Affymetrix microarrays. RNA samples from four undifferentiated iPSCs (ABP 05, ABP06, ABP11 and ABP12) were also included.

**Fig 4:**
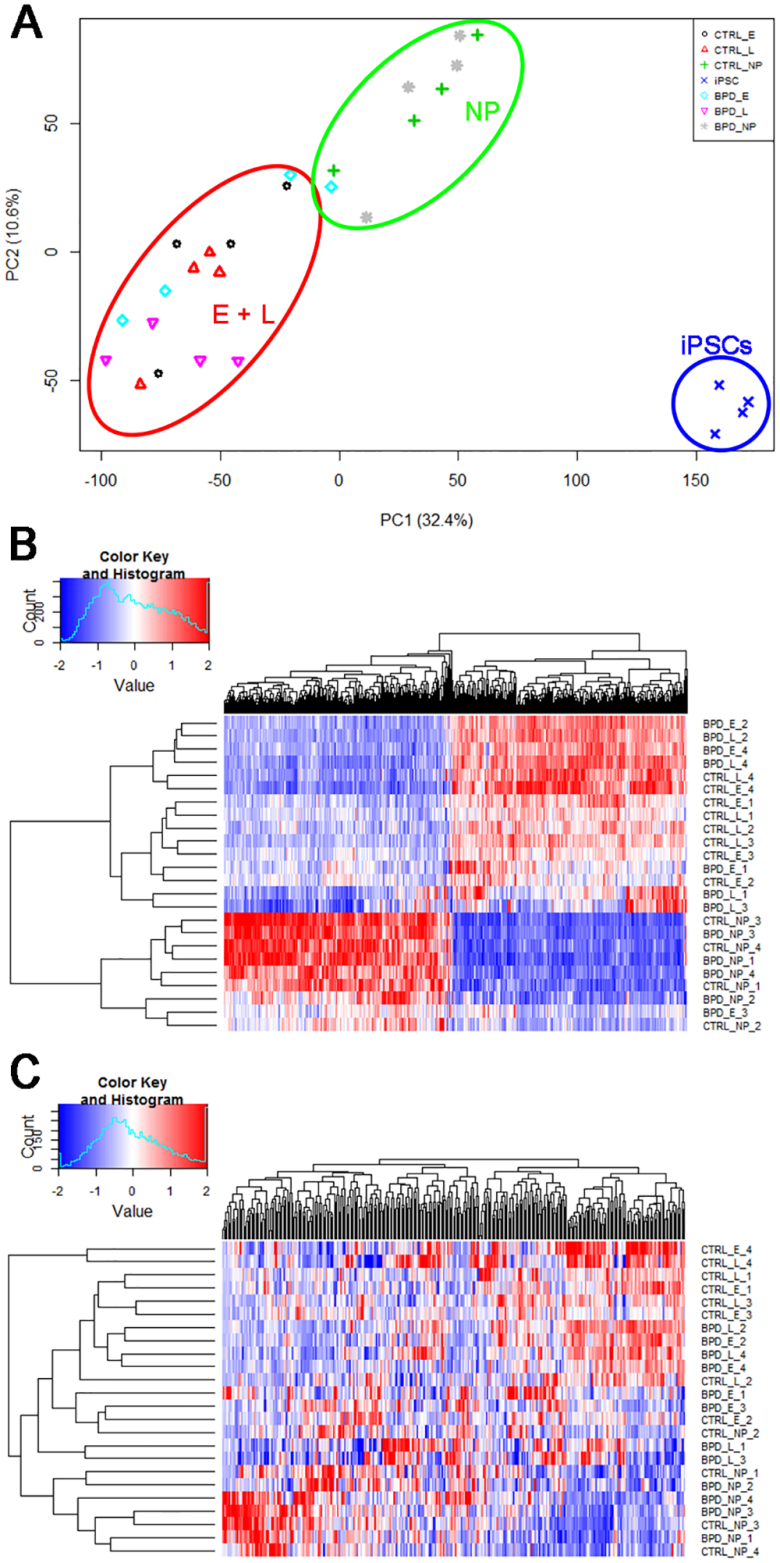
Microarray data analysis. A. Principal Component Analysis (PCA) plot shows three distinct clusters: iPSCs, NPs and E plus L neurons as outlined with blue, green and red lines, respectively. The symbols represent undifferentiated iPSCs (blue x), control NP (green cross; +), control E (black circle; ○), control L (red triangle; Δ) and BPD NP (grey asterisk; *), BPD E (turquoise diamond; ♢) and BPD L (pink inverted triangle; ▽). B. Clustered heat map of differentially expressed genes in both control and BPD neurons demonstrates that the genes are clustered closely for E and L neurons regardless of disease status and separate from NPs and iPSCs. Horizontal axis shows genes used for clustering in the S2 Table and vertical axis shows sample clustering. C. Changes in expression of genes associated with axonal guidance (Ingenuity pathway analysis) shown as a clustered heat map show that the expression of genes associated with axonal guidance was higher in E and L neurons than in NPs. Horizontal axis shows genes used for clustering as listed in S3 Table.

To analyze global gene expression changes associated with different stage of differentiation, Principal Component Analysis (PCA) was performed on the microarray data. As shown in Fig 4A, PCA identified three distinct clusters corresponding to iPSCs, NPs and neurons at either E or L stage with no differences between BPD and controls at any stage of cellular differentiation.

Subsequently, DEGs between NP and E neurons, as well as NP and L neurons, were identified and processed for hierarchical clustering of gene expression, which illustrated close assembly of E and L neuron expression profiles separating from those of iPSCs and NPs (Fig 4B). BPD_E_3 was an apparent outlier. Without technical replicates it is not possible to determine if this variation comes from culture effects, clonal variation or inherent differences between patients. There was no clear segregation of BPD and control cells. This was consistent with the lack of morphological differences between BPD and control cells.

To annotate functions to genes regulated during differentiation, enrichment analysis of DEGs was performed. The GO analysis of DEGs demonstrated that the progressive differentiation from NP to E and L neurons was associated primarily with genes involved in cell cycle regulation and neurogenesis in both BPD and control populations (S4 and S5 Tables). There was a robust alteration in the expression of genes associated with neuronal differentiation and specification as exemplified by the axonal guidance signaling pathway (defined by Ingenuity Pathway Analysis) upon differentiation from iPSC to NP to E and L neurons (Fig 4C). Importantly, the patterns of gene expression were similar in RNAs isolated from BPD and control donors. Collectively, these data demonstrate that our differentiation paradigm was robust and BPD iPSCs differentiated similarly to the control iPSCs.

### Differentially Regulated Genes (DEGs) between BPD and control cells

Having confirmed similar pattern and time course of differentiation between BPD and control iPSCs, analysis of DEGs between BPD and controls at each time point was performed. Interestingly, there were no significant differences between BPD and controls at either NP or E stages of differentiation. However, L neurons from BPD cases compared to controls showed differential expressions for 697 probes, which corresponded to 324 genes. Out of the 324 genes, 292 genes showed upregulation in BPDs compared to their sibling controls while only 32 genes were downregulated. The complete list of DEGs between BPD and control L neurons is provided in Table 1.

**Table 1:** List of differentially expressed genes (DEGs) in control and BPD L neurons.

**Upregulated in BPD neurons**						
PTPN3	ZNF669	APOPT1	SMCHD1	DHX57	COL4A3BP	BDP1
CLTC	SNRPN	SPTAN1	ATXN3	ZCCHC6	ZNF274	NFYC
GSK3B	CSNK1G1	FUBP3	UBE2W	KMT2C	MINK1	KLHL24
C9orf64	ZNF800	OCIAD1	CECR1	LINC00537	TRIM66	AGAP4
TMEM87A	NFX1	G6PC2	CPEB4	MALAT1	RBM39	PLD6
KMT2A	CCZ1B	DICER1-AS1	DBT	CROCCP2	REEP2	MGEA5
HCG18	PDCD6	SLC22A3	PAPD4	PWRN2	SCFD1	KIF13A
MGC57346	LETMD1	BRWD1	FNBP4	FCRL2	GREB1L	LMBRD2
HNRNPD	CRB3	UBE2H	KANSL1	ZNF654	GPATCH2L	SLC23A2
CMBL	RIOK3	PDIA2	SLC25A27	ZXDC	PKN2	ZBTB11
CLASP2	CSNK1A1	FNBP1	NFATC3	JAK2	PIK3C2A	SYMPK
DYNC1H1	SBNO1	SFTPB	DIP2A	DIO2-AS1	MTX3	VPS53
C10orf12	LOC102546299	L3MBTL1	FKSG49	ZMYM5	POLR1B	TTC5
MINOS1P1	HIST1H2BD	NPAS3	PHF20L1	NLN	STAG2	CEP95
ZNF335	TMEM161B	UBE2D3	DUSP16	C9orf84	RAB21	CAPN1
TRAF4	TRAF3IP2-AS1	HAUS2	NDFIP2	TCP11L2	NKTR	N4BP2L2
PPP2R5C	WDR11	CCAR1	CWF19L2	WDR41	CYLD	COX17
CTTN	NFASC	TTBK2	FGFR1	ZNF160	MDM2	MRPS5
SCD5	FGFR1OP2	LOC151121	SLC35E1	KPNA5	ANKRD10-IT1	OSBP
CCDC90B	GTF2A2	TXNL1	ITGB8	SPG7	MYLK3	ZBTB7A
LRCH3	TRMT13	FXR1	PPM1A	TMEM33	SIK3	CKMT2-AS1
TRPS1	ACVR1B	PHF3	MEG3	SLC6A15	ZNF493	CNTFR-AS1
KLF12	CBFA2T2	VPS13C	ABI2	KIDINS220	AZI2	C4orf29
DIP2B	RBM5	RBM33	SLC25A36	AHI1	ZBTB38	MACF1
USP34	LOC100272216	SUPT20H	TTC37	ERP44	ATP6V1H	ZCCHC11
RFX7	AFF4	RASAL2	LRRIQ1	PHKB	ZFYVE16	TGDS
RAB18	KIF21A	ZC3H7B	CLEC4F	ANKRD12	LOC728093	CAMSAP2
SNRPA1	SCAF11	GALNT2	CYHR1	FAM169A	NCAPH2	RBBP6
IFNL1	ZNF791	MGC12488	PDXDC1	ZNF518A	ELOVL5	CLOCK
STRAP	LINC01007	NFAT5	STK32A	PAAF1	PNISR	KIAA1522
TMEM91	CNOT4	GRM5	CDK13	SGSM1	PGF	PCMTD1
ICA1	DLGAP4	PTEN	TMF1	CRYZL1	FOXK1	LOC101926996
MYO6	VPS8	LPXN	TTC17	GNG2	ANKRD13A	PGAP1
PRKAA1	URGCP	AGFG1	MZT2B	HELB	AUTS2	ATP9A
KLHDC10	SEPT2	ALMS1	NDST4	CAPN3	C11orf80	GOPC
SBF2	GOSR1	MORN4	SART1	CEP290	GAD1	RFPL3S
EEA1	RBM14	ARHGAP5	FAM132A	BOD1L1	ATF7IP	LOC100507311
FCF1	ZNF577	GOLGA4	LOC283045	KIAA0754	PIP5K1A	CLINT1
RAB12	FAM161B	MTMR9	EPG5	ZCCHC7	PPP1R3E	NBR1
FAM208B	UACA	NAALAD2	PPWD1	RHOQ	TTLL3	MED6
CCNT1	SERPINB6	FLJ41455	STK38	CRIPAK	MON2	CSAD
TMEM67	PPA2	ZNF207	MKLN1	WDR61		
**Downregulated in BPD neurons**						
PRCD	C3orf70	CDKN2C	MVK	RAB22A	TTL	TRAM2-AS1
VPS13B	SLC6A20	MKI67	NEK1	LYRM7	GPR125	MYO1D
ZADH2	NOL12	ALMS1	TMEM44	LOC728705	ACADM	KCTD15
MCOLN1	PBK	PMEPA1	GAB1	SCN4B	ELOVL2	ASPH
C6orf203	FAM105A	PARD3	RPTOR			

Total DEGs in L neurons between BPD and control (combined up and down regulated DEGs) were annotated by multiple pathway analysis tools. GO analysis performed on all DEGs suggested that the group differences reflect enrichment of genes involved in metabolic processes, including macromolecule and cellular RNA metabolism and biosynthesis pathways. Also identified were pathways involving protein transport including post-Golgi vesicle mediated transport and Golgi to plasma membrane protein transport (Table 2). Many of the pathways found for the total DEGs were seen when up and down regulated DEGs were analyzed separately eg cilium assembly, metabolic processes (Table 2). Although the number of down-regulated genes was small (32 genes), GO analysis revealed cell cycle pathways (Table 2).

**Table 2:** TOP 10 list of GO pathway analysis of DEGs between BPD and control in L neurons.

**TOTAL**						
**Ranking**	**GOBPID**	**Term**	**Size of GO Term**	**Matched**	**P value**	**FDR**
1	GO:0006892	post-Golgi vesicle-mediated transport	75	8	4.89E-05	0.001623
2	GO:0042384	cilium assembly	78	8	6.49E-05	0.001623
3	GO:0044260	cellular macromolecule metabolic process	6283	139	0.000115	0.001911
4	GO:0006355	regulation of transcription, DNA-dependent	2724	71	0.0002	0.002024
5	GO:0008152	metabolic process	8966	183	0.000202	0.002024
6	GO:0051252	regulation of RNA metabolic process	2804	72	0.000281	0.002346
7	GO:0015031	protein transport	1202	37	0.000525	0.00333
8	GO:0043001	Golgi to plasma membrane protein transport	23	4	0.000629	0.00333
9	GO:0032774	RNA biosynthetic process	3087	76	0.000664	0.00333
10	GO:0010556	regulation of macromolecule biosynthetic process	3046	75	0.000731	0.00333
**UP-REGULATED**						
**Ranking**	**GOBPID**	**Term**	**Size of GO Term**	**Matched**	**P value**	**FDR**
1	GO:0006355	regulation of transcription, DNA-dependent	2724	69	1.76E-05	0.000431
2	GO:0006892	post-Golgi vesicle-mediated transport	75	8	2.31E-05	0.000431
3	GO:0051252	regulation of RNA metabolic process	2804	70	2.44E-05	0.000431
4	GO:0032774	RNA biosynthetic process	3087	74	5.26E-05	0.000652
5	GO:0010556	regulation of macromolecule biosynthetic process	3046	73	6.15E-05	0.000652
6	GO:0051171	regulation of nitrogen compound metabolic process	3326	77	0.000115	0.00099
7	GO:0031326	regulation of cellular biosynthetic process	3171	74	0.000131	0.00099
8	GO:0071704	organic substance metabolic process	8524	160	0.000165	0.001017
9	GO:0090304	Nucleic acid metabolic process	4168	91	0.000173	0.001017
10	GO:0042384	cilium assembly	78	7	0.000227	0.00112
**DOWN- REGULATED**						
**Ranking**	**GOBPID**	**Term**	**Size of GO Term**	**Matched**	**P value**	**FDR**
1	GO:0007049	cell cycle	1508	8	0.00265	0.008484
2	GO:0055117	regulation of cardiac muscle contraction	66	2	0.005583	0.008484
3	GO:0035383	thioester metabolic process	68	2	0.005916	0.008484
4	GO:0051283	negative regulation of sequestering of calcium ion	75	2	0.007155	0.008484
5	GO:0010991	negative regulation of SMAD protein complex assembly	5	1	0.008484	0.008484
6	GO:0031585	regulation of inositol 1,4,5-trisphosphate-sensitive calcium-release channel activity	5	1	0.008484	0.008484
7	GO:0033539	fatty acid beta-oxidation using acyl-CoA dehydrogenase	5	1	0.008484	0.008484
8	GO:0045329	carnitine biosynthetic process	5	1	0.008484	0.008484
9	GO:0086067	AV node cell to bundle of His cell communication	5	1	0.008484	0.008484
10	GO:1901339	regulation of store-operated calcium channel activity	5	1	0.008484	0.008484

GO pathway analysis was performed on all DEGs identified in L neurons and up- and down-regulated DEGs separately.

GeneGo analysis of total DEGs and upregulated DEGs (S6 Table) revealed potential involvement of protein transport as well as the Wnt signaling pathway. Due to the small sample size together with the finding that <2 matched genes were observed for each GeneGo pathway, conclusions could not be drawn for the down-regulated DEGS using this particular analysis tool (data not shown). From Ingenuity Pathway Analysis (IPA), receptor signaling pathways were enriched between BPD and control L neurons (Table 3). Similar results were obtained when analysis was performed on the up-regulated DEGS only (Table 3). As for GeneGo, an analysis of down-regulated DEGs was not appropriate for IPA. KEGG pathway analysis on DEGs was also performed (S7 Table) and supported the results from the other analyses, identifying receptor-mediated signaling, RNA metabolism, and protein trafficking as major processes potentially dysregulated in BPD. Interestingly, many of the enriched pathways involved GSK3β, which has been implicated as a therapeutic target of lithium [42], the first-line treatment for BPD [43].

**Table 3:** TOP 10 list of IPA pathway analysis of DEGs between BPD and control in L neurons.

**TOTAL**		
**RANK**	**Pathway**	**Hits Symbol**
1	Integrin Signaling	GSK3B,FNBP1,PIK3C2A,CAPN1,CTTN,ITGB8,MYLK3,PTEN,CAPN3,ARHGAP5,RHOQ
2	Insulin Receptor Signaling	GSK3B,JAK2,PIK3C2A,GAB1,PTEN,RPTOR,RHOQ
3	Amyloid Processing	GSK3B,CSNK1A1,CAPN1,CAPN3
4	HER-2 Signaling in Breast Cancer	GSK3B,PIK3C2A,MDM2,ITGB8,PARD3
5	PI3K/AKT Signaling	GSK3B,JAK2,PPP2R5C,MDM2,ITGB8,GAB1,PTEN
6	ILK Signaling	GSK3B,FNBP1,PIK3C2A,PPP2R5C,ITGB8,PGF,PTEN,RHOQ
7	Taurine and Hypotaurine Metabolism	GAD1,CSAD
8	Role of NFAT in Regulation of the Immune Response	GSK3B,CSNK1G1,CSNK1A1,NFATC3,PIK3C2A,KPNA5,NFAT5,GNG2
9	Hypoxia Signaling in the Cardiovascular System	UBE2H,UBE2D3,MDM2,PTEN
10	IL-4 Signaling	NFATC3,JAK2,PIK3C2A,NFAT5
**UP-REGULATED**		
**RANK**	**Pathway**	**Hits Symbol**
1	Integrin Signaling	GSK3B,FNBP1,PIK3C2A,CAPN1,CTTN,ITGB8,MYLK3,PTEN,CAPN3,ARHGAP5,RHOQ
2	Amyloid Processing	GSK3B,CSNK1A1,CAPN1,CAPN3
3	ILK Signaling	GSK3B,FNBP1,PIK3C2A,PPP2R5C,ITGB8,PGF,PTEN,RHOQ
4	Role of NFAT in Regulation of the Immune Response	GSK3B,CSNK1G1,CSNK1A1,NFATC3,PIK3C2A,KPNA5,NFAT5,GNG2
5	Taurine and Hypotaurine Metabolism	GAD1,CSAD
6	Hypoxia Signaling in the Cardiovascular System	UBE2H,UBE2D3,MDM2,PTEN
7	IL-4 Signaling	NFATC3,JAK2,PIK3C2A,NFAT5
8	IL-17A Signaling in Airway Cells	GSK3B,JAK2,PIK3C2A,PTEN
9	Melanoma Signaling	PIK3C2A,MDM2,PTEN
10	PI3K/AKT Signaling	GSK3B,JAK2,PPP2R5C,MDM2,ITGB8,PTEN

IPA pathway analysis of total DEGs and up-regulated DEGs performed independently shows enrichment of receptor signaling pathways.

### Confirmational qRT-PCR

**Fig 5:**
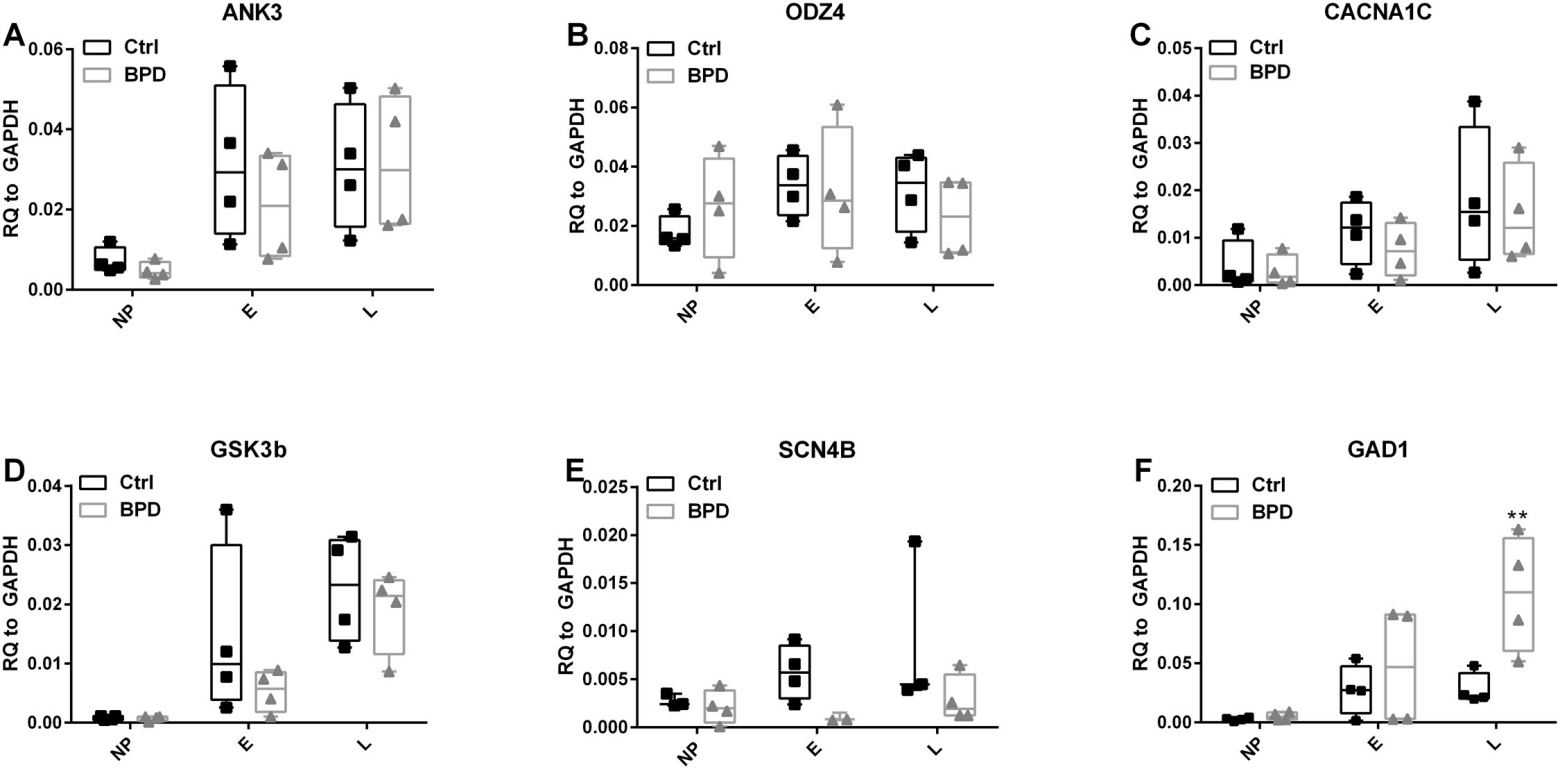
Confirmational quantitative RT-PCR. RNA samples collected during differentiation at NP, E and L neuron stages for microarray studies were analyzed by quantitative RT-PCR. Genes previously implicated to be involved with BPD from genomic studies were evaluated: ANK3, ODZ4 and CACNA1C were analyzed (A-C) and did not show differences in expression at any stage. GSK3B did not show significant differences in BPD and control at any stage (D). SCN4B showed trends of down regulation both in E and L neurons (E), but did not meet the statistical significance. Expression of GAD1 was upregulated in L of BPD compare to control. The box on the plots represents the 25 and 75 percentiles. The horizontal bar within the box represents the median. Individual data points are represented by triangles (BPD) or squares (Controls). n = 4 except for SCN4B NP and L controls and E BPD where n = 3. Two-way ANOVA was performed and Bonferroni’s Multiple Comparison post-hoc test was done. ** p < 0.01 when compared to control.

To validate DEGs identified from microarray studies, expression of selected genes was measured by qRT-PCR from an independent aliquot of RNA to that used for the microarray analysis. We first studied the expression of previously reported candidate genes from the literature on genetic studies of BPD: ANK3, ODZ4 and CACNA1C [44–46]. Consistent with the microarray data, qRT-PCR failed to show alterations in the expression of these genes in BPD versus control samples (Fig 5A–5C). We also examined the expression of mRNA encoding GSK3β, a molecule implicated in the mechanism of therapeutic response to lithium [42, 47], which showed small changes (Fold change of BPD_L/CTRL_L = 1.6) in the microarray, but failed to show a significant difference by qRT-PCR (Fig 5D). Voltage gated type IV sodium channel beta subunit, SCN4B, was identified as one of the highly down-regulated genes by the microarray in L neurons of BPD samples (fold change = -14.6). Expression of SCN4B mRNA also showed a decrease by qRT-PCR, but did not reach statistical significance due to high variability between samples (Fig 5E). Glutamate decarboxylase 1 (GAD1) showed 2.5-fold increase in the microarray analysis of L neurons, which was confirmed by qRT-PCR (Fig 5F).

Samples from neuronal differentiation of 2 additional BPD and 2 control iPSC lines, were also analyzed by qRT-PCR. These lines were derived from fibroblasts from the same Old Amish population isolate (Coriell Catalog ID: GM05926, GM05934, GM05904, GM05930) using the methods described above. These cases had the same diagnostic reliability, and for the BPD patients, lithium responsiveness. Inclusion of data from these samples did not change the overall expression profiles of candidate genes assessed (data not shown).

## Discussion

In this study, we report the successful generation and characterization of iPSC lines from 4 affected and 4 unaffected BPD individuals from an Old Order Amish pedigree and their differentiation into NPs and neurons. We purposely chose subjects with an unequivocal diagnosis of BPD and their healthy unaffected first-degree relatives, the presence or absence of affective disorders being ascertained longitudinally over the years and ratings being carried out blindly and conceded by a psychiatric review board. Consistent with literature, differentiation of the iPSCs into NPs and neurons was accompanied by changes in the expression of signature markers of pluripotency, neuroectoderm and neurons. This is the largest cohort reported to date for iPSC generation from a population isolate with a high prevalence of BPD. By selecting samples from a culturally and genetically isolated Amish population, we anticipated to increase the probability of identifying molecular changes associated with BPD.

One major objective of this study was to conduct global gene expression analysis on iPSC-derived neurons of control and BPDs in order to generate hypotheses related to BPD biology. To ensure that the differentiation paradigm employed in this study was robust, we used the Affymetrix microarray platform to analyze RNA from iPSCs, NPs and E and L neurons. Consistent with signature gene markers analyzed by qPCR, the data subjected to Principal Component Analysis clearly demonstrated three isolated clusters of gene expression corresponding to iPSCs, NPs and neurons, validating the differentiation method we employed. Moreover, the Gene Ontology and Ingenuity Pathway analyses on DEGs associated with progressive differentiation of iPSCs confirmed the expected enrichment of genes and pathways associated with cell cycle regulation, neurogenesis and axon guidance as cells progressed from iPSCs to NPs to E or L neurons. These data are largely consistent with the recent report by Chen et al. [21] who also implemented withdrawal of growth factors to induce commitment of iPSCs to neuronal differentiation and observed enrichment of neuron specific markers in the transcriptome of neurons versus undifferentiated iPSCs.

Critically, we observed that BPD and control iPSCs differentiated similarly to NPs and neurons, thereby paving the way to identify DEGs between controls and BPDs at a given stage of differentiation of iPSCs. This is in contrast to Madison et al [22], who observed a deficit in the proliferative characteristics of NPs as well as in neuronal differentiation ability in their BPD lines. Although we presented one iPSC cell line/donor for 4 donors in the current study, we did confirm the NP differentiation capacity of 2 additional lines from each of the 4 BPD donors and 3 lines from 2 additional affected family members (data not shown). Chen et al [21] did not report a lack of differentiation capacity in their BPD iPSCs.

Analysis of microarray data to identify DEGs between BPD and control samples failed to detect significant changes at NP or E stage neurons. It should be noted that we applied criteria that we believed would minimize false positives in our identification of DEGs (see Materials and Methods). The ability to identify DEGs only in the L neuronal population indicates that the maturity level of neurons may be an important factor to consider for transcriptomic studies of iPSC-derived neurons. This contention is supported by our observations (Fig 3 and S5 Table) that expression of key synaptic markers and proteins involved in the regulation of neuronal function progressively increase in expression from E to L neurons. The timing of neuronal differentiation from iPSCs has been intensively debated in the field with a longer differentiation duration reported to have clearer impact on neuronal function [48].

We conducted qRT-PCR analyses for 2 genes whose expression was highly altered in the microarray analysis. Consistent with microarray data, the expression of GAD1, the gene encoding glutamate acid decarboxylase, a key enzyme for gamma amino butyric acid (GABA) synthesis, showed a significant increase in BPD L neurons. The decrease in the expression of SCN4B (encodes the beta subunit of voltage gated Type IV sodium channel) observed by microarray analysis was also seen as a reduction in L neurons of BPD cases by qRT-PCR, but this finding failed to reach statistical significance. SCN4B is known to regulate neuronal activity and appears to be especially important during brain development [49]. Changes in the expression of both of these genes have been reported in postmortem tissues from schizophrenia [50,51]. Thus the increase in GAD1 concurrently with a decrease in SCN4B indicates that BPD may be associated with an imbalance of excitatory and inhibitory neurotransmission in developing neurons. Interestingly, postmortem data on autopsied hippocampus indicate a decrease in GAD1 mRNA in BPD patients [11]. These data raise the question whether the increase in GAD1 mRNA in developing neurons differentiated from iPS cells is a compensatory response. Of note, Madison et al. [22] also found GAD1 to be differentially regulated between BPD and control CXCR4+ NPs.

We studied by qRT-PCR three other genes, ANK3, CACNA1C and ODZ4 that have been implicated by genetic studies of BPD [44–46]. As with the microarray study, the mRNA for these genes did not show significant differences between BPD and controls in L neurons by qRT-PCR. Similar to our findings at 4 weeks of neural differentiation, Madison et. al. [22] did not detect differences in expression of these genes at 6 weeks using the PsychGene NanoString probe set. Bavamian et al [52] did show decreases in ANK3 gene expression in BPD lines compared to controls at 2 and 8 weeks of neural differentiation but not at 4 or 6 weeks.

The DEGs between BPD and controls identified by microarray analysis were subjected to multiple pathway analysis to understand their potential functional significance. Gene Ontology (GO) indicated that the DEGs reflected alterations in biosynthetic and metabolic pathways for several macromolecules, including RNAs. Defective RNA metabolism contributes to a variety of neurologic diseases, especially motor neuron diseases [53]. Importantly, our findings are consistent with the GO analysis of the microarray study of postmortem brain samples of BPD and controls, where Chen et al [15] reported changes in protein and macromolecule metabolic processes. In addition, these investigators also observed changes in markers of synaptic transmission. Taken together, changes in pre-mRNA processing or RNA editing of neuronal receptors and ion channels that regulate synaptic activity may be important phenotypes associated with BPD.

GO pathway analysis revealed that DEGs between BPD and controls were enriched in protein transport as well as post-Golgi transport pathways. Interestingly, GeneGo analysis also identified clathrin-coated vesicle as well ER-to-Golgi processes. Chen et al [15] identified protein transport in their pathway analyses of DEGs in post-mortem BPD brains. This suggests that disruption of vesicular trafficking and protein transport may play a role in BPD and is supported by other recent genomic and proteomic findings implicating the clathrin interactome in psychiatric disorders including BPD [54].

Another enriched pathway identified by GO analysis of total,up and down regulated DEGs implicated differential regulation in cilium assembly in BPD neurons. Cilium, a microtubule enriched cellular structure, appears to play a key role in developmental signaling cascades, especially associated with hedgehog (Hh) signaling molecules [55]. A report by Breunig et. al. [56] showed that primary cilia are required for Sonic Hedgehog (Shh) pathways and that mutations in ciliary components can lead to phenotypes similar to the phenotypes of Hh/Shh pathway dysregulation, leading to many different pathologies including cognitive deficits and behavioral disorders [55]. A recent report demonstrated that disruption in Shh signaling underlies Ellis-van Creveld dwarfism syndrome, which has a protective effect against development of BPD [57]. This observation, together with our identification of cilium assembly as a pathway modulated in BPD, suggests that disruption in cilial assembly and associated developmental signaling cascade may contribute to BPD pathogenesis.

In multiple pathway analyses, signaling pathways were found in the top 10 rankings. GeneGo analysis of the up-regulated DEGs in BPD L neurons identified the wnt signaling pathway including GSK3β. Ingenuity Pathway Analysis of DEGs showed enrichment of receptor-mediated signaling pathways. One observation was that virtually all of the top pathways identified were associated with GSK3β. This may be due to over-prevalence of GSK3β in IPA pathways; however, GSK3β has been suggested to play a role in the therapeutic effects of lithium [42,47]. Lithium is the treatment of choice for preventing episodes of affective disorder in bipolar subjects and specifically in these Amish families, where good responsiveness and compliance has been documented over decades [24]. In particular, the BPD patients in our study were all good lithium responders based on medication histories recorded longitudinally (see Methods section). Quantitative RT-PCR analysis however failed to show a difference in GSK3β mRNA expression. Since the activity of this enzyme is largely regulated by posttranslational phosphorylation [58,59], future studies of BPD-derived iPS neurons might explore GSK3β protein expression and phosphorylation, as well as the response to lithium. Other components identified in our IPA pathway analysis should also be evaluated.

Chen et al. [21] conducted microarray analysis of 3 BPD and 3 control iPSCs and neurons derived from these iPSCs. Although our GO annotations, derived from DEGs in iPSCs versus neurons, are similar to those reported by Chen et al, we did not see a major overlap in DEGs in BPD neurons compared to control between the two studies. A key reason for this underlying discrepancy may be the fact that Chen et al. conducted microarray analysis on neurons differentiated for 8 weeks, whereas ours were differentiated for 4 weeks. Alternatively the genetic heterogeneity of BPD revealed by GWAS [45,60] and, more recently, WGS [8,44] studies may underlie this lack of overlap. Our samples were derived from an Old Order Amish pedigree, whereas Chen et al. studied iPSCs reprogrammed from Caucasian donors from unrelated patients and controls. Madison et al [22] reported seeing differential gene expression as early as the NP stage between BPD and control population. This discrepancy might be due to their use of an enriched NP population (CXCR4+) or the different analytical platforms employed. RNA-seq has a wider dynamic range and is more sensitive than microarray which may also have contributed to the identification of DEGs at this time point. In their study of iPSC-derived neurons differentiated for 6 weeks, Madison et al. found 44 DEGs between BPD and controls using the 352 custom PsychGene Nanostring probe set. We saw no overlap between their DEGs and the DEGs that we identified between BPD and control samples in our L neuronal population.

In our study, we randomly picked one iPSC line per donor on the basis of recent studies demonstrating that the genetic background of individual donors has greater influence on the transcriptome than line-to-line differences or the reprogramming method used [61]. However, we cannot exclude the significance of line-to-line variability in modeling diseases using iPSCs. Even though the variability between lines might not be as significant as the variability coming from each donor, inclusion of multiple lines might be beneficial to reduce technical errors in future experiments.

In summary, our study represents the first report of characterizing iPS cell lines and iPSC-derived neurons derived from a genetic isolate, the Old Order Amish. Having applied a robust differentiation protocol, we were able to assess molecular mechanisms associated with BPD by microarray analysis on neurons derived from the Amish pedigree. The differentially expressed gene expression data lead us to hypothesize that the alterations in RNA metabolic processes, protein trafficking and receptor-mediated signaling contribute to BPD biology. The 4 BPD and 4 control lines from the Amish pedigree now offers us the opportunity to test these hypotheses in order to gain further insights into the biology associated with this devastating disease.

## Supporting Information

S1 Fig Schematic representation of iPSC differentiation into NPs and neurons and times of sample collection. IPSCs maintained in feeder-free system were dissociated, allowed to form EBs in AggreWell800 for 5 days, and further differentiated after plating prior to collection of NPs. NPs were expanded further before starting neuronal differentiation by growth factor withdrawal. RNA samples were collected from iPSCs, NPs, and from early (E) and late (L) neurons, defined as 2 and 4 weeks after growth factor withdrawal from NPs, respectively. Representative images of each of the stages of differentiation are shown to the right. iPSCs were stained for Oct4, Neural rosettes for Pax6, Neural progenitors for Pax6 (green), Nestin (red) and nuclei (Hoechst33342), and L neurons for MAP2 (red) and β-Tubulin III (green). Photomicrographs of EBs and E neurons are shown.
(TIF)

S2 Fig Characterization of iPSCs. Successful reprogramming of fibroblasts into iPSCs has been shown by RT-PCR of pluripotent stem cell markers DNMT3b, hTERT, NANOG, OCT4, REX1 and SOX2. Inactivation of viral particles was also confirmed by RT-PCR using Sendai-virus specific primers set (SEV) (A). The iPS cells were shown to be positive for alkaline phosphatase. Expression of pluripotent protein markers was also shown by immunocytochemistry for OCT4, Nanog, Tra1-60, SSEA-3 and SSEA-4 (white bar = 100μm). Fibroblasts surrounding human iPS colonies serve as internal negative controls for IHC staining. (B). The ability of iPSCs to give rise to three germ layers was confirmed by forming EBs and testing expression of mesodermal (FLK2, GATA2), endodermal (AFP, GATA4) and ectodermal (PAX6, N-CAM) markers by RT-PCR (C).
(TIF)

S3 Fig Quantitative RT-PCR of ion channel gene expression in NPs and neurons. RNA samples, collected from NPs, E and L neurons, were analyzed for ion channel protein expression. Each bar represents mean ± standard error (n = 4). One-way ANOVA was performed and Bonferroni’s Multiple Comparison post-hoc test was done. * p < 0.05, ** p < 0.01 compared to NP. The changes were not statistically significant in some cases, but the trend for increases in expression was observed.
(TIFF)

S1 Table List of TaqMan probes used for quantitative RT-PCR. Gene expression was measured by quantitative RT-PCR using (A) Custom Primer probes designed by Primer3 with indicated annealing temperatures, or (B) pre-designed TaqMan^®^ probes (Life Technologies).
(DOCX)

S2 Table List of genes included for generation of clustering heat map in Fig 4B. The genes that were identified to be differentially expressed in neurons (E and L) from NP are listed in this table. The genes are listed in the order generated by hierarchical clustering in the heat map.
(DOCX)

S3 Table List of genes included for generation of clustering heat map of axonal guidance. The table lists genes of axonal guidance defined from IPA, which were used for the generation of the heat map in Fig 4C.
(DOCX)

S4 Table GO pathway analysis of DEGs between NP and E. DEGs from microarray data were analyzed for BPD and control lines independently by GO pathway analysis. Both BPD and control showed enrichment in genes associated with cell proliferation, neurogenesis, and axon guidance.
(DOCX)

S5 Table GO pathway analysis of DEGs between NP and L. DEGs from microarray data were analyzed for BPD and control lines independently by GO pathway analysis. Both BPD and control showed enrichment of in cell proliferation, neurogenesis, and axon guidance.
(DOCX)

S6 Table Top 10 list of GeneGo analysis of total and up-regulated DEGs between BPD and control in L neurons. GeneGo analysis of total and up- regulated DEGs in L neurons was performed and the top 10 ranked pathways listed.
(DOCX)

S7 Table Top 10 list of KEGG analysis of DEGs between BPD and control in L neurons. KEGG analysis on DEGs in L neurons was performed and top10 ranked pathways listed.
(DOCX)
