# Supplementary figures and images for: Transcriptomic Analysis of Induced Pluripotent Stem Cells Derived from Patients with Bipolar Disorder from an Old Order Amish Pedigree
Source: PLoS One. 2015 Nov 10;10(11):e0142693. doi: 10.1371/journal.pone.0142693 (PMC4640865; doi:10.1371/journal.pone.0142693)

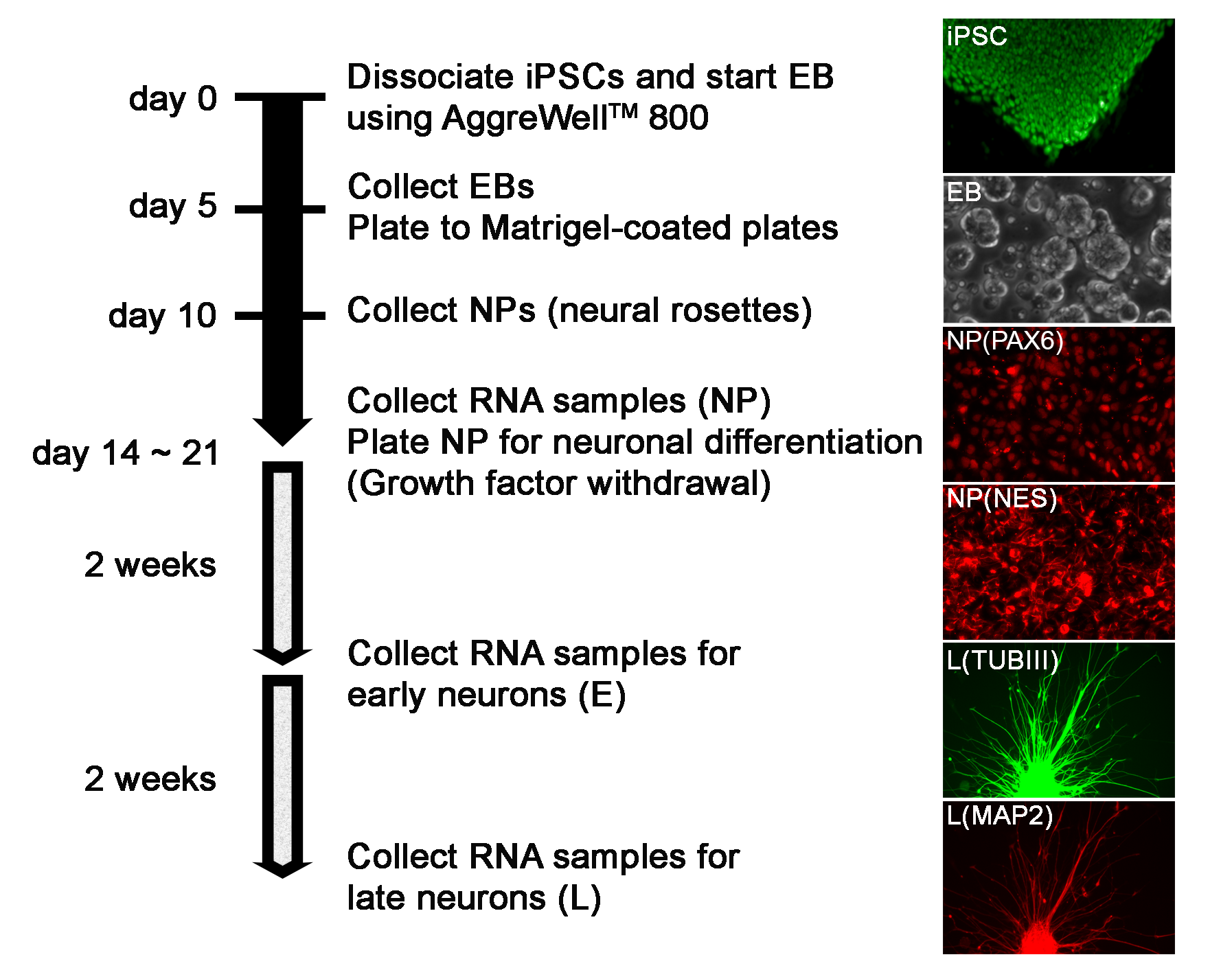

Supplement: S1 Fig — IPSCs maintained in feeder-free system were dissociated, allowed to form EBs in AggreWell800 for 5 days, and further differentiated after plating prior to collection of NPs. NPs were expanded further before starting neuronal differentiation by growth factor withdrawal. RNA samples were collected from iPSCs, NPs, and from early (E) and late (L) neurons, defined as 2 and 4 weeks after growth factor withdrawal from NPs, respectively. Representative images of each of the stages of differentiation are shown to the right. iPSCs were stained for Oct4, Neural rosettes for Pax6, Neural progenitors for Pax6 (green), Nestin (red) and nuclei (Hoechst33342), and L neurons for MAP2 (red) and β-Tubulin III (green). Photomicrographs of EBs and E neurons are shown. (TIF) [file pone.0142693.s001.tif]

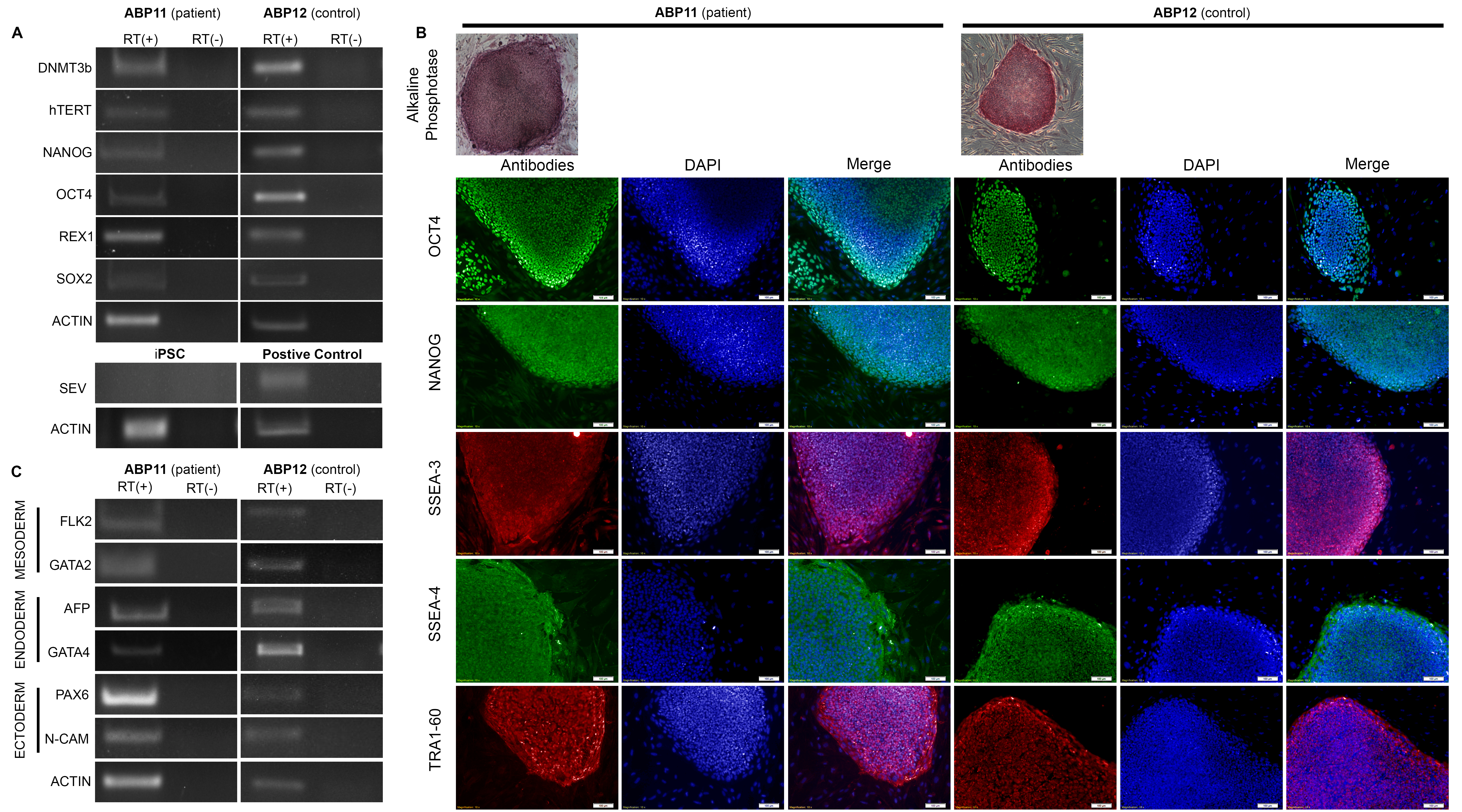

Supplement: S2 Fig — Successful reprogramming of fibroblasts into iPSCs has been shown by RT-PCR of pluripotent stem cell markers DNMT3b, hTERT, NANOG, OCT4, REX1 and SOX2. Inactivation of viral particles was also confirmed by RT-PCR using Sendai-virus specific primers set (SEV) (A). The iPS cells were shown to be positive for alkaline phosphatase. Expression of pluripotent protein markers was also shown by immunocytochemistry for OCT4, Nanog, Tra1-60, SSEA-3 and SSEA-4 (white bar = 100μm). Fibroblasts surrounding human iPS colonies serve as internal negative controls for IHC staining. (B). The ability of iPSCs to give rise to three germ layers was confirmed by forming EBs and testing expression of mesodermal (FLK2, GATA2), endodermal (AFP, GATA4) and ectodermal (PAX6, N-CAM) markers by RT-PCR (C). (TIF) [file pone.0142693.s002.tif]
