# Supplementary material for: Transcriptomic Analysis of Induced Pluripotent Stem Cells Derived from Patients with Bipolar Disorder from an Old Order Amish Pedigree
Source: PLoS One. 2015 Nov 10;10(11):e0142693. doi: 10.1371/journal.pone.0142693 (PMC4640865; doi:10.1371/journal.pone.0142693)
Supplement: S1 Table — Gene expression was measured by quantitative RT-PCR using (A) Custom Primer probes designed by Primer3 with indicated annealing temperatures, or (B) pre-designed TaqMan® probes (Life Technologies). (DOCX) [file pone.0142693.s004.docx]

1. **Custom Primer Probes**

| **Gene** | **Primer Forward** | **Primer Reverse** | **Annealing Temp** |
| --- | --- | --- | --- |
| PAX6 | tctaatcgaagggccaaatg | tgtgagggctgtgtctgttc | 57^o^C |
| FLK1 | agtgatcggaaatgacactgga | gcacaaagtgacacgttgagat | 63^o^C |
| GATA2 | gcaacccctactatgccaacc | cagtggcgtcttggagaag | 58^o^C |

1. **Commercial Primer Probes**

| **Gene** | **Alternative Name** | **Cat#** |
| --- | --- | --- |
| POU5F1 | OCT4 | Hs00999634_gH |
| PAX6 |  | Hs00240871_m1 |
| NESTIN |  | Hs04187831_g1 |
| FOXG1 |  | Hs01850784_s1 |
| GLI3 |  | Hs00609233_m1 |
| TUBB3 | Tubulin, class iii | Hs00801390_s1 |
| MAP2 |  | Hs00258900_m1 |
| DLX2 |  | Hs00269993_m1 |
| CTIP2 | BCL11B | Hs00256257_m1 |
| VGLUT1 | SLC17A7 | Hs00220404_m1 |
| GAD1 |  | Hs01065893_m1 |
| DARPP-32 | PPP1R1B | Hs00259967_m1 |
| SYP | SYNAPTOPHYSIN | Hs00300531_m1 |
| SYN1 | SYNAPSIN | Hs00199577_m1 |
| ANK3 |  | Hs00241738_m1 |
| ODZ4 |  | Hs01008080_m1 |
| GSK3b |  | Hs01047719_m1 |
| SCN4b |  | Hs03681025_m1 |
| CACNA1B | Cav2.2 | Hs01053090_m1 |
| CACNA1C | Cav1.2 | Hs00167681_m1 |
| CACNA1D | Cav1.3 | Hs01073321_m1 |
| CACNA1E | Cav2.3 | Hs00167789_m1 |
| GABRA1 |  | Hs00971228_m1 |
| GABRB2 |  | Hs00241451_m1 |
| GABRG2 |  | Hs00168093_m1 |
| GRIA1 | GLUR1; AMPA1 | Hs00181348_m1 |
| GRIA2 | GLUR2; AMPA2 | Hs00181331_m1 |
| GRIN1 | NMDAR1 | Hs00609557_m1 |
| GRIN2A | NMDAR2A | Hs00168219_m1 |
| GRIN2B | NMDAR2B | Hs00168230_m1 |
| GRIN2C | NMDAR2C | Hs01016626_m1 |
| GRIN2D | NMDAR2D | Hs00181352_m1 |
