# Supplementary material for: Transcriptomic Analysis of Induced Pluripotent Stem Cells Derived from Patients with Bipolar Disorder from an Old Order Amish Pedigree
Source: PLoS One. 2015 Nov 10;10(11):e0142693. doi: 10.1371/journal.pone.0142693 (PMC4640865; doi:10.1371/journal.pone.0142693)
Supplement: S2 Table — The genes that were identified to be differentially expressed in neurons (E and L) from NP are listed in this table. The genes are listed in the order generated by hierarchical clustering in the heat map. (DOCX) [file pone.0142693.s005.docx]

| 1 | SLC16A1 | RBPMS | DPPA4 | CTSC | KCNK5 | LYN | GNAS-AS1 | RNF135 | ST6GAL1 | B3GNT2 |
| --- | --- | --- | --- | --- | --- | --- | --- | --- | --- | --- |
| 11 | PAWR | DAZAP1 | CYBA | TRIP10 | SLC50A1 | LOC100049716 | HEATR1 | KIAA0020 | DNAJC2 | SWAP70 |
| 21 | ARPC1B | MCCC2 | HMGA1 | ZNF215 | IMPA2 | TBC1D1 | LYAR | ORC2 | SNAP23 | DNAJC15 |
| 31 | NUFIP1 | MGST1 | ZNF217 | TGIF1 | C1orf85 | SHPK | DNMBP | GALNT12 | DCP2 | DUSP23 |
| 41 | DSC2 | CDC14B | ACSS3 | ARAP3 | SLC31A1 | YES1 | HELLS | NUP205 | MCM3 | PARPBP |
| 51 | TMEM243 | GEMIN4 | PTGIS | METTL13 | NAA15 | MGME1 | MIS18A | AURKA | POLR3K | BMPR1A |
| 61 | CCDC86 | AIF1L | KIF20B | EPHB4 | REST | RANGRF | DDX10 | LGALS8 | HK2 | ASPH |
| 71 | RASL12 | SMARCD2 | PLSCR1 | RREB1 | MAN2B1 | C1RL | EPB41L2 | EML4 | ARID3A | TTLL4 |
| 81 | RAB27A | NET1 | STK3 | PDE5A | MYLK | HDGF | EED | PCTP | CDC7 | MCM8 |
| 91 | IGF2BP1 | TEX30 | WEE1 | UGDH | TEAD2 | ASPM | KIF14 | ORC6 | CBX2 | LMNB1 |
| 101 | GINS4 | DTL | SKP2 | BRIP1 | FAM64A | HAT1 | CYYR1 | CCDC138 | PRKD3 | PLK4 |
| 111 | TICRR | SMC1A | TPM1 | ABCC4 | GJC1 | PXDN | GPC3 | HIP1 | C1orf106 | BCAT1 |
| 121 | MAP3K1 | HMGA2 | CEP152 | ELAVL1 | FANCI | CA3 | TMEM135 | ATP11A | B3GALNT2 | SLC25A13 |
| 131 | DIAPH3 | PKP4 | CTDSPL | GJA1 | GPD2 | RBMS1 | MFAP2 | DEPDC1 | SPDL1 | LXN |
| 141 | TMEM216 | MEIS3P1 | HLA-DPB1 | SLC43A3 | WIBG | RPAP3 | RECQL | MIS18BP1 | ECT2 | PKN3 |
| 151 | HAUS8 | FXN | NCAPH | GULP1 | HEPH | LPP | LOXL2 | GPX8 | SLC45A3 | FANCB |
| 161 | AVEN | PGM2 | TUBD1 | SNRNP48 | ZDHHC20 | STIL | C1orf109 | DCAF13 | PGRMC2 | SHMT1 |
| 171 | PLA2G3 | TM6SF2 | PAICS | DIMT1 | BCL2L12 | FANCD2 | HAUS6 | ANLN | MASTL | MTFR2 |
| 181 | EXOSC3 | RAD51 | MCM4 | OIP5 | CENPK | CKS1B | RAD51AP1 | SPAG5 | PTBP1 | TCF3 |
| 191 | CTNNAL1 | LARP4 | GGA2 | ANAPC16 | PIK3C2A | CCDC150 | SYNJ2 | CDC14A | KIF16B | EIF4E |
| 201 | ABRACL | C14orf39 | G2E3 | CDC25A | MAP2K6 | NSMCE4A | GTF3C2 | PHF13 | ALDH3A2 | DLAT |
| 211 | LIN28B | LIN28A | SALL4 | LUZP1 | ARID3B | FBXO11 | APLF | AP1S2 | ITSN1 | RFC5 |
| 221 | ENSA | UACA | PSME4 | SLC26A6 | FARP1 | PLIN2 | RABGAP1L | VASH2 | RNF41 | SPHK1 |
| 231 | KIRREL | TPM2 | ROR2 | C17orf96 | NOTCH2 | CREB3L2 | EMP2 | DERA | YAP1 | COL9A1 |
| 241 | SELENBP1 | IFT74 | GALM | MALT1 | SLC35G1 | PIP5K1B | CLIC1 | CYFIP1 | ITGA5 | MAGT1 |
| 251 | CPS1 | SNX18 | NR2C1 | PTPN13 | ERF | SLC25A37 | SP1 | MTBP | ZNF516 | TMEM123 |
| 261 | ANP32A | CCDC160 | PRTG | GLI3 | FBLN7 | F2RL2 | KLF3 | NRIP1 | WHSC1 | NEDD4 |
| 271 | CREM | ITPRIPL2 | HS2ST1 | IKBIP | UHRF1 | CMTM3 | PTX3 | METRN | PCGF5 | LRCH3 |
| 281 | PCSK1N | RAB6B | ATP1B1 | LIN7A | HIST3H2A | CNTN1 | CELF6 | CLASP2 | PSD2 | NAPB |
| 291 | STMN4 | GNG3 | SS18L1 | NDRG4 | LYST | HIST2H2BE | VAMP2 | ZNF25 | FAM126B | SYNJ1 |
| 301 | PIK3CA | UBR3 | TRIM52 | HCFC2 | ZBTB41 | ASB8 | AAK1 | RALGAPA1 | DIRAS2 | ZNF540 |
| 311 | CPEB4 | HEATR5B | ACSL6 | GNL1 | NOL4 | ANKS1B | DPY19L2P2 | PPFIA2 | GPR137C | DUSP8 |
| 321 | GATS | ATP6V1G2 | GDAP1L1 | TERF2IP | RUFY3 | CLTB | WDR47 | TAOK3 | PARP6 | SMAP2 |
| 331 | NOVA2 | KLHL42 | EVL | MVB12B | ATL1 | GPM6A | COMMD3 | STX7 | PPM1K | GNAI1 |
| 341 | ITFG1 | AGPAT3 | DIP2C | TAPT1 | ANKH | PPP1R21 | LBH | AP3B2 | ANKRD50 | SOX2-OT |
| 351 | ZNF827 | CDKN2C | FGF9 | LOC100288310 | RUNX1T1 | ST18 | RPS6KA2 | POU2F2 | ACAP3 | BRSK2 |
| 361 | KCNH8 | KLHL35 | CRMP1 | RGMB | DPF1 | RNF165 | LCOR | PCBP4 | SLC44A5 | FBRSL1 |
| 371 | SOX4 | KIF5A | MYT1 | WDFY3 | TP53INP2 | THSD7A | GDAP1 | ELAVL2 | DCX | NEFL |
| 381 | SLC4A8 | ELAVL4 | SYT4 | STMN2 | KIF5C | SRGAP3 | CELF3 | APLP1 | MAP1B | GSK3B |
| 391 | FNIP2 | TET2 | DCC | SRRM4 | NFASC | C20orf194 | REM2 | CECR6 | PACS2 | ACTL6B |
| 401 | NSG1 | RUNDC3A | RTN1 | NCAM1 | INA | FAM13C | PKIA | CSRNP3 | NOVA1 | MARK1 |
| 411 | CADPS | SVOP | ZC2HC1A | CTIF | PCSK2 | NRG3 | DYNC1H1 | SYT1 | RPS6KL1 | RAB39B |
| 421 | CELSR3 | MTMR9 | ATCAY | PGM2L1 | DISP2 | NMNAT2 | DCLK1 | CD200 | FBXL16 | AKAP6 |
| 431 | KIAA1211 | CEP170 | BMPR2 | NBEA | RTN2 | KIAA0930 | SLC24A3 | CNR1 | BICD1 | PARD6A |
| 441 | CPLX2 | PGBD5 | ABHD5 | KIF1A | GSE1 | CAMK2N2 | RGS2 | POU6F1 | SHANK2 | GLTSCR1L |
| 451 | FAM57B | DNAJB5 | ABCG1 | BCL11A | FAM220A | FEZ1 | PTP4A1 | MALAT1 | RAB2B | DNAJC18 |
| 461 | TRPC3 | ACVR1B | GPR56 | EIF1 | TRIM36 | FSD1L | ARHGAP21 | PHF20L1 | SPAG9 | KATNAL1 |
| 471 | FAM127A | RND3 | DYNC1LI2 | ELAVL3 | GDI1 | NACAD | RIIAD1 | SLC43A2 | SUN2 | SLC16A14 |
| 481 | TUBB3 | TAGLN3 | MAP6 | SMARCD3 | DCAF5 | CHD3 | MEAF6 | DCTN3 | PNMA2 | STAM2 |
| 491 | LINC00674 | JUN | RUFY2 | MLLT4 | STX12 | RAB6A | NAPG | TOX | FBXW7 | ZNF536 |
| 501 | PLK3 | BTBD10 | ITGB8 | BRWD1 | TRAPPC6B | CSNK1E | SMIM8 | ZMIZ2 | AK1 | STMN3 |
| 511 | GNG4 | HN1 | GSTA4 | ACTR1A | PDZD4 | KCNQ2 | KIFAP3 | ABAT | CDKN2D | WSB2 |
| 521 | GNG2 | FAM49A | GAP43 | ST8SIA2 | KLC1 | DLL3 | C14orf132 | GADD45G | CYFIP2 | KIF1B |
| 531 | SETBP1 | GPC2 | PLEKHA6 | INSM1 | UG0898H09 | ACSL1 | LOC646241 | KLF7 | B4GALNT1 | RGAG4 |
| 541 | ACYP2 | MANEAL | C3orf14 | COQ9 | ASCL1 | CAMK1D | SHD | UBE2Q2 | KLF13 | PDE1A |
| 551 | SRGAP1 | NFIB | C9orf91 | JAK1 | SMARCA2 | TRAK2 | LTBP3 |  |  |  |
