# Supplementary material for: Transcriptomic Analysis of Induced Pluripotent Stem Cells Derived from Patients with Bipolar Disorder from an Old Order Amish Pedigree
Source: PLoS One. 2015 Nov 10;10(11):e0142693. doi: 10.1371/journal.pone.0142693 (PMC4640865; doi:10.1371/journal.pone.0142693)
Supplement: S3 Table — The table lists genes of axonal guidance defined from IPA, which were used for the generation of the heat map in Fig 4C. (DOCX) [file pone.0142693.s006.docx]

| 1 | PIK3CD | EPHA1 | PDGFA | GNA14 | SEMA6A | TUBA4A | PRKCH | RAC2 | PIK3CB | PRKCI |
| --- | --- | --- | --- | --- | --- | --- | --- | --- | --- | --- |
| 11 | ERBB2 | SEMA4B | ITGA6 | RRAS2 | VASP | SEMA3F | SEMA4D | PRKCQ | ITGB5 | ARPC5L |
| 21 | KRAS | RND1 | GNB1L | GNA13 | GNA12 | GNG7 | CRKL | NFATC1 | ROCK1 | PRKD3 |
| 31 | PAK4 | PIK3R4 | EPHB4 | FZD6 | TUBG1 | ARPC1B | PFN1 | EIF4E | MYL12A | NRAS |
| 41 | VEGFA | ADAM10 | PTK2 | SHC1 | PIK3C2A | PTPN11 | MAPK3 | SEMA3A | FZD10 | WNT9A |
| 51 | ITGA4 | MKNK1 | ITSN1 | MICAL1 | EFNA4 | GNG5 | ITGB1 | RHOA | ARPC1A | NTF3 |
| 61 | CXCL12 | ABL1 | MYL12B | ARPC2 | FZD8 | FZD5 | FZD7 | SEMA4C | PRKAG2 | UNC5B |
| 71 | PXN | PLCB3 | ITGA2B | MAP2K1 | RRAS | PLXND1 | GNA15 | BDNF | SEMA4G | PAK1 |
| 81 | SEMA4A | ADAM8 | PPP3R1 | WASL | MAP2K2 | MYLPF | MYL1 | MYL4 | ITGA1 | NGF |
| 91 | SEMA5A | NCK2 | ITGB2 | ITGA8 | EPHA6 | MYL10 | ITGA10 | ABLIM3 | PLCB4 | RASSF5 |
| 101 | GNAL | ROBO1 | PIK3R2 | UNC5D | ADAM23 | PRKAR1B | PRKCB | PPP3CA | HERC2 | ITGB3 |
| 111 | ITGAM | WNT8A | PFN4 | ITGAL | FARP2 | WNT6 | PRKACA | ITGB6 | PPP3R2 | EGF |
| 121 | NRP2 | ITGB4 | WNT16 | EPHA8 | ADAM21 | LIMK1 | SEMA6B | PRKCG | MAG | PIK3R6 |
| 131 | ARHGEF15 | PIK3R5 | RAP1A | EPHB6 | SUFU | WNT10B | WNT4 | BMP7 | WNT8B | WNT2B |
| 141 | BMP2 | EPHA7 | WNT1 | PRKCA | ADAM19 | MYL9 | SMO | FZD4 | NGEF | ITGAV |
| 151 | ITGA7 | GLI3 | EFNA5 | WNT5B | STK36 | EFNB2 | PRKD1 | SDC2 | NFATC3 | ITGAE |
| 161 | ROCK2 | EPHA4 | CRK | WNT7B | EFNA2 | FZD2 | GLI2 | HKR1 | NFATC4 | GNB3 |
| 171 | AKT2 | NFAT5 | EFNB1 | ADAM17 | EPHA2 | ADAM29 | ADAM28 | ITGA2 | ADAM18 | ATM |
| 181 | VEGFC | BMP5 | WNT3 | GNG11 | UNC5C | WIPF1 | GNB4 | RGS3 | DOCK1 | ITGA5 |
| 191 | TUBB6 | SRGAP2 | GNG12 | PDGFD | EFNB3 | MYL6 | PFN2 | MYL5 | ACTR3 | ARPC4 |
| 201 | SDCBP | RAP1B | MAPK1 | GNAI3 | ACTR2 | GNAI2 | NCK1 | RAC1 | ARPC3 | RHOD |
| 211 | MYL6B | TUBA1C | TUBA1B | CFL1 | TUBB | GNAO1 | PAK7 | CDC42 | EPHA5 | GNAZ |
| 221 | ARHGEF7 | ARHGEF12 | ABLIM2 | ADAM22 | SEMA3E | PRKACB | ROBO2 | SEMA6D | NTRK3 | LIMK2 |
| 231 | MRAS | PIK3R1 | CDK5 | PPP3CB | PAK3 | GNAQ | PRKAR2B | PLXNA1 | EPHB1 | ABLIM1 |
| 241 | ARHGEF11 | ADAM11 | GRB2 | GNG4 | RTN4 | TUBB2A | TUBB3 | GNG2 | DCC | L1CAM |
| 251 | SRGAP3 | KLC1 | EFNA3 | ROBO3 | NGFR | EPHB3 | NTRK2 | PLXNA3 | GNG3 | PIK3CA |
| 261 | ITGB8 | PLXNA2 | EPHA3 | SOS1 | WNT7A | AKT1 | NTNG1 | LRRC4C | SRGAP1 | PPP3CC |
| 271 | SEMA4F | GNAI1 | PIK3C3 | SLIT3 | PLCB1 | SLIT2 | SOS2 | FZD3 | HRAS | GNA11 |
| 281 | ARPC5 | TUBB1 | GNAS | GSK3B | PIK3R3 | GNB1 | DPYSL2 | FYN | DPYSL5 | PTCH1 |
| 291 | PIK3C2B | RAF1 | NTN1 | BMP1 | SHH | HHIP | ITGA11 | RASA1 | KALRN | WNT5A |
| 301 | ITGA9 | NRP1 | FZD1 | ADAM12 | PLXNB2 | BAIAP2 | CXCR4 | GNB2 | PRKAR2A | PDGFC |
| 311 | AKT3 | CFL2 | PAK2 | PRKAR1A | SEMA3C | ADAM9 | SEMA3D | PGF | TUBB2B | EPHB2 |
| 321 | TUBA1A | PLXNC1 | WAS | EPHA10 | BMP8B | GIT1 | PDGFB | PAK6 | LINGO1 | SHANK2 |
| 331 | GNB5 | ARHGEF6 | GLIS2 | PRKAG1 | BMP10 | RAC3 | RTN4R |  |  |  |
