# Supplementary material for: Transcriptomic Analysis of Induced Pluripotent Stem Cells Derived from Patients with Bipolar Disorder from an Old Order Amish Pedigree
Source: PLoS One. 2015 Nov 10;10(11):e0142693. doi: 10.1371/journal.pone.0142693 (PMC4640865; doi:10.1371/journal.pone.0142693)
Supplement: S4 Table — DEGs from microarray data were analyzed for BPD and control lines independently by GO pathway analysis. Both BPD and control showed enrichment in genes associated with cell proliferation, neurogenesis, and axon guidance. (DOCX) [file pone.0142693.s007.docx]

| **Ranking** | **GOBPID** | **Term** | **Pvalue** | **FDR** | **ExpCount** | **Count** | **Size** |
| --- | --- | --- | --- | --- | --- | --- | --- |
|  | **CONTROL NP vs. E** | | | | | | |
| 1 | GO:0048285 | organelle fission | 7.68E-26 | 1.83E-23 | 33.33654 | 102 | 373 |
| 2 | GO:0000278 | mitotic cell cycle | 1.28E-24 | 1.52E-22 | 37.20876 | 107 | 430 |
| 3 | GO:0051301 | cell division | 4.10E-22 | 3.25E-20 | 31.77713 | 93 | 359 |
| 4 | GO:0051325 | interphase | 3.87E-16 | 2.30E-14 | 35.39214 | 88 | 396 |
| 5 | GO:0000279 | M phase | 5.89E-16 | 2.81E-14 | 38.09693 | 92 | 434 |
| 6 | GO:0048812 | neuron projection morphogenesis | 3.14E-15 | 1.24E-13 | 49.60262 | 108 | 555 |
| 7 | GO:0048667 | cell morphogenesis involved in neuron differentiation | 7.44E-14 | 2.53E-12 | 49.15575 | 104 | 550 |
| 8 | GO:0007067 | mitosis | 1.96E-13 | 5.84E-12 | 13.51916 | 45 | 156 |
| 9 | GO:0030182 | neuron differentiation | 9.50E-13 | 2.51E-11 | 85.44163 | 151 | 956 |
| 10 | GO:0032990 | cell part morphogenesis | 1.35E-12 | 3.08E-11 | 59.88064 | 116 | 670 |
| 11 | GO:0007017 | microtubule-based process | 1.42E-12 | 3.08E-11 | 22.08671 | 59 | 253 |
| 12 | GO:0010564 | regulation of cell cycle process | 4.41E-12 | 8.75E-11 | 30.08497 | 71 | 340 |
| 13 | GO:0030030 | cell projection organization | 7.85E-12 | 1.37E-10 | 81.59854 | 143 | 913 |
| 14 | GO:0000236 | mitotic prometaphase | 8.03E-12 | 1.37E-10 | 7.418049 | 30 | 83 |
| 15 | GO:0006260 | DNA replication | 1.12E-11 | 1.78E-10 | 23.77351 | 60 | 266 |
| 16 | GO:0048468 | cell development | 3.18E-11 | 4.73E-10 | 130.1287 | 202 | 1456 |
| 17 | GO:0007018 | microtubule-based movement | 9.41E-11 | 1.32E-09 | 14.8361 | 43 | 166 |
| 18 | GO:0045786 | negative regulation of cell cycle | 7.24E-10 | 9.57E-09 | 39.6821 | 80 | 444 |
| 19 | GO:0007411 | axon guidance | 8.55E-10 | 1.07E-08 | 30.92344 | 67 | 346 |
| 20 | GO:0000075 | cell cycle checkpoint | 2.35E-09 | 2.79E-08 | 18.10686 | 46 | 204 |
|  | **BPD NP vs. E** | | | | | | |
| 1 | GO:0000278 | mitotic cell cycle | 7.39E-22 | 2.00E-19 | 54.19468 | 128 | 430 |
| 2 | GO:0048285 | organelle fission | 8.92E-21 | 1.21E-18 | 48.07737 | 116 | 373 |
| 3 | GO:0051325 | interphase | 3.24E-16 | 2.93E-14 | 51.04192 | 111 | 396 |
| 4 | GO:0006271 | DNA strand elongation involved in DNA replication | 7.96E-16 | 5.32E-14 | 4.382387 | 25 | 34 |
| 5 | GO:0051301 | cell division | 9.82E-16 | 5.32E-14 | 45.78329 | 102 | 359 |
| 6 | GO:0048858 | cell projection morphogenesis | 6.14E-14 | 2.50E-12 | 84.94098 | 153 | 659 |
| 7 | GO:0007017 | microtubule-based process | 6.47E-14 | 2.50E-12 | 32.22609 | 77 | 253 |
| 8 | GO:0031175 | neuron projection development | 1.11E-13 | 3.76E-12 | 79.55953 | 145 | 621 |
| 9 | GO:0000075 | cell cycle checkpoint | 4.51E-13 | 1.36E-11 | 31.45007 | 74 | 244 |
| 10 | GO:0016043 | cellular component organization | 1.72E-12 | 4.67E-11 | 335.9242 | 445 | 2731 |
| 11 | GO:0000279 | M phase | 4.32E-12 | 1.06E-10 | 55.22308 | 107 | 434 |
| 12 | GO:0007067 | mitosis | 2.31E-11 | 5.22E-10 | 19.96428 | 52 | 158 |
| 13 | GO:0022008 | neurogenesis | 4.14E-11 | 8.62E-10 | 130.0026 | 201 | 1017 |
| 14 | GO:0007010 | cytoskeleton organization | 6.16E-11 | 1.19E-09 | 101.6972 | 165 | 789 |
| 15 | GO:0000236 | mitotic prometaphase | 1.51E-10 | 2.72E-09 | 10.69818 | 34 | 83 |
| 16 | GO:0000722 | telomere maintenance via recombination | 6.88E-10 | 1.14E-08 | 3.351237 | 17 | 26 |
| 17 | GO:0007411 | axon guidance | 7.17E-10 | 1.14E-08 | 44.59723 | 86 | 346 |
| 18 | GO:0045786 | negative regulation of cell cycle | 9.84E-10 | 1.48E-08 | 57.22882 | 103 | 444 |
| 19 | GO:0000084 | S phase of mitotic cell cycle | 1.36E-09 | 1.94E-08 | 17.78734 | 45 | 138 |
| 20 | GO:0032201 | telomere maintenance via semi-conservative replication | 1.44E-09 | 1.96E-08 | 3.09345 | 16 | 24 |
