# Supplementary material for: Transcriptomic Analysis of Induced Pluripotent Stem Cells Derived from Patients with Bipolar Disorder from an Old Order Amish Pedigree
Source: PLoS One. 2015 Nov 10;10(11):e0142693. doi: 10.1371/journal.pone.0142693 (PMC4640865; doi:10.1371/journal.pone.0142693)
Supplement: S5 Table — DEGs from microarray data were analyzed for BPD and control lines independently by GO pathway analysis. Both BPD and control showed enrichment of in cell proliferation, neurogenesis, and axon guidance. (DOCX) [file pone.0142693.s008.docx]

| **Ranking** | **GOBPID** | **Term** | **Pvalue** | **FDR** | **ExpCount** | **Count** | **Size** |
| --- | --- | --- | --- | --- | --- | --- | --- |
|  | **CONTROL NP vs. L** | | | | | | |
| 1 | GO:0000278 | mitotic cell cycle | 7.39E-22 | 2.00E-19 | 54.19468 | 128 | 430 |
| 2 | GO:0048285 | organelle fission | 8.92E-21 | 1.21E-18 | 48.07737 | 116 | 373 |
| 3 | GO:0051325 | Interphase | 3.24E-16 | 2.93E-14 | 51.04192 | 111 | 396 |
| 4 | GO:0006271 | DNA strand elongation involved in DNA replication | 7.96E-16 | 5.32E-14 | 4.382387 | 25 | 34 |
| 5 | GO:0051301 | cell division | 9.82E-16 | 5.32E-14 | 45.78329 | 102 | 359 |
| 6 | GO:0048858 | cell projection morphogenesis | 6.14E-14 | 2.50E-12 | 84.94098 | 153 | 659 |
| 7 | GO:0007017 | microtubule-based process | 6.47E-14 | 2.50E-12 | 32.22609 | 77 | 253 |
| 8 | GO:0031175 | neuron projection development | 1.11E-13 | 3.76E-12 | 79.55953 | 145 | 621 |
| 9 | GO:0000075 | cell cycle checkpoint | 4.51E-13 | 1.36E-11 | 31.45007 | 74 | 244 |
| 10 | GO:0016043 | cellular component organization | 1.72E-12 | 4.67E-11 | 335.9242 | 445 | 2731 |
| 11 | GO:0000279 | M phase | 4.32E-12 | 1.06E-10 | 55.22308 | 107 | 434 |
| 12 | GO:0007067 | Mitosis | 2.31E-11 | 5.22E-10 | 19.96428 | 52 | 158 |
| 13 | GO:0022008 | Neurogenesis | 4.14E-11 | 8.62E-10 | 130.0026 | 201 | 1017 |
| 14 | GO:0007010 | cytoskeleton organization | 6.16E-11 | 1.19E-09 | 101.6972 | 165 | 789 |
| 15 | GO:0000236 | mitotic prometaphase | 1.51E-10 | 2.72E-09 | 10.69818 | 34 | 83 |
| 16 | GO:0000722 | telomere maintenance via recombination | 6.88E-10 | 1.14E-08 | 3.351237 | 17 | 26 |
| 17 | GO:0007411 | axon guidance | 7.17E-10 | 1.14E-08 | 44.59723 | 86 | 346 |
| 18 | GO:0045786 | negative regulation of cell cycle | 9.84E-10 | 1.48E-08 | 57.22882 | 103 | 444 |
| 19 | GO:0000084 | S phase of mitotic cell cycle | 1.36E-09 | 1.94E-08 | 17.78734 | 45 | 138 |
| 20 | GO:0032201 | telomere maintenance via semi-conservative replication | 1.44E-09 | 1.96E-08 | 3.09345 | 16 | 24 |
|  | **BPD NP vs. L** | | | | | | |
| 1 | GO:0000278 | mitotic cell cycle | 1.76E-28 | 5.38E-26 | 189.3362 | 323 | 718 |
| 2 | GO:0071840 | cellular component organization or biogenesis | 6.61E-26 | 1.01E-23 | 1090.71 | 1342 | 4116 |
| 3 | GO:0048285 | organelle fission | 6.02E-24 | 6.12E-22 | 98.84229 | 189 | 373 |
| 4 | GO:0051301 | cell division | 1.28E-18 | 9.79E-17 | 122.6916 | 209 | 463 |
| 5 | GO:0051325 | Interphase | 1.48E-16 | 9.05E-15 | 104.9371 | 180 | 396 |
| 6 | GO:0007017 | microtubule-based process | 5.97E-15 | 3.04E-13 | 66.61426 | 124 | 253 |
| 7 | GO:0000279 | M phase | 2.20E-14 | 9.58E-13 | 114.0366 | 186 | 434 |
| 8 | GO:0000236 | mitotic prometaphase | 3.47E-14 | 1.32E-12 | 21.9944 | 55 | 83 |
| 9 | GO:0022402 | cell cycle process | 5.07E-13 | 1.72E-11 | 100.6438 | 165 | 393 |
| 10 | GO:0006271 | DNA strand elongation involved in DNA replication | 1.11E-12 | 3.38E-11 | 9.009753 | 29 | 34 |
| 11 | GO:0045786 | negative regulation of cell cycle | 5.64E-12 | 1.56E-10 | 117.6568 | 183 | 444 |
| 12 | GO:0007010 | cytoskeleton organization | 4.71E-11 | 1.20E-09 | 209.0793 | 290 | 789 |
| 13 | GO:0007052 | mitotic spindle organization | 5.64E-10 | 1.32E-08 | 9.274745 | 27 | 35 |
| 14 | GO:0007059 | chromosome segregation | 2.33E-09 | 5.07E-08 | 28.80266 | 58 | 109 |
| 15 | GO:0000084 | S phase of mitotic cell cycle | 2.83E-09 | 5.76E-08 | 36.569 | 69 | 138 |
| 16 | GO:0007067 | Mitosis | 5.56E-09 | 1.06E-07 | 41.25795 | 75 | 158 |
| 17 | GO:0010564 | regulation of cell cycle process | 6.49E-09 | 1.17E-07 | 85.86244 | 133 | 326 |
| 18 | GO:0032990 | cell part morphogenesis | 7.71E-09 | 1.31E-07 | 177.5451 | 243 | 670 |
| 19 | GO:0030030 | cell projection organization | 8.44E-09 | 1.35E-07 | 241.9384 | 317 | 913 |
| 20 | GO:0032201 | telomere maintenance via semi-conservative replication | 9.38E-09 | 1.43E-07 | 6.359825 | 20 | 24 |
