# Supplementary material for: Transcriptomic Analysis of Induced Pluripotent Stem Cells Derived from Patients with Bipolar Disorder from an Old Order Amish Pedigree
Source: PLoS One. 2015 Nov 10;10(11):e0142693. doi: 10.1371/journal.pone.0142693 (PMC4640865; doi:10.1371/journal.pone.0142693)
Supplement: S6 Table — GeneGo analysis of total and up- regulated DEGs in L neurons was performed and the top 10 ranked pathways listed. (DOCX) [file pone.0142693.s009.docx]

| **TOTAL** | | | | | | |
| --- | --- | --- | --- | --- | --- | --- |
| RANK | Pathway | Size of Pathway | Matched | P value | FDR | Matched.Symbol |
| 1 | Delta508-CFTR traffic / ER-to-Golgi in CF | 6 | 2 | 6 | 2 | GOPC,RHOQ |
| 2 | Normal wtCFTR traffic / ER-to-Golgi | 6 | 2 | 6 | 2 | GOPC,RHOQ |
| 3 | Beta-alanine metabolism/ Rodent version | 8 | 2 | 8 | 2 | GAD1,CSAD |
| 4 | Beta-alanine metabolism | 8 | 2 | 8 | 2 | GAD1,CSAD |
| 5 | Non-genomic (rapid) action of Androgen Receptor | 27 | 3 | 27 | 3 | GSK3B,MDM2,PTEN |
| 6 | AKT signaling | 28 | 3 | 28 | 3 | MDM2,PTEN,GAB1 |
| 7 | FGF2-dependent induction of EMT | 12 | 2 | 12 | 2 | FGFR1,GAB1 |
| 8 | PIP3 signaling in cardiac myocytes | 31 | 3 | 31 | 3 | PTEN,GAB1,PARD3 |
| 9 | WNT signaling pathway. Part 1. Degradation of beta-catenin in the absence WNT signaling | 13 | 2 | 13 | 2 | GSK3B,CSNK1A1 |
| 10 | wtCFTR and delta508 traffic / Clathrin coated vesicles formation (norm and CF) | 13 | 2 | 13 | 2 | CLTC,MYO6 |
| **UP-REGULATED** | | | | | | |
| RANK | Pathway | Size of Pathway | Matched | P value | FDR | Matched.Symbol |
| 1 | Delta508-CFTR traffic / ER-to-Golgi in CF | 6 | 2 | 1.41E-05 | 0.004646 | GOPC,RHOQ |
| 2 | Normal wtCFTR traffic / ER-to-Golgi | 6 | 2 | 1.41E-05 | 0.004646 | GOPC,RHOQ |
| 3 | Beta-alanine metabolism/ Rodent version | 8 | 2 | 3.89E-05 | 0.006423 | GAD1,CSAD |
| 4 | Beta-alanine metabolism | 8 | 2 | 3.89E-05 | 0.006423 | GAD1,CSAD |
| 5 | Non-genomic (rapid) action of Androgen Receptor | 27 | 3 | 9.10E-05 | 0.012031 | GSK3B,MDM2,PTEN |
| 6 | Clathrin-coated vesicle cycle | 57 | 4 | 0.000146 | 0.013487 | CLTC,MYO6,GOSR1,EEA1 |
| 7 | WNT signaling pathway. Part 1. Degradation of beta-catenin in the absence WNT signaling | 13 | 2 | 0.000192 | 0.013487 | GSK3B,CSNK1A1 |
| 8 | wtCFTR and delta508 traffic / Clathrin coated vesicles formation (norm and CF) | 13 | 2 | 0.000192 | 0.013487 | CLTC,MYO6 |
| 9 | GABA-A receptor life cycle | 13 | 2 | 0.000192 | 0.013487 | CLTC,DYNC1H1 |
| 10 | wtCFTR and delta508-CFTR traffic / Generic schema (norm and CF) | 33 | 3 | 0.000204 | 0.013487 | CLTC,GOPC,RHOQ |
