# Supplementary material for: Transcriptomic Analysis of Induced Pluripotent Stem Cells Derived from Patients with Bipolar Disorder from an Old Order Amish Pedigree
Source: PLoS One. 2015 Nov 10;10(11):e0142693. doi: 10.1371/journal.pone.0142693 (PMC4640865; doi:10.1371/journal.pone.0142693)
Supplement: S7 Table — KEGG analysis on DEGs in L neurons was performed and top10 ranked pathways listed. (DOCX) [file pone.0142693.s010.docx]

| RANK | Pathway | Size of Pathway | Matched | P value | FDR | Matched.Symbol |
| --- | --- | --- | --- | --- | --- | --- |
| 1 | Biosynthesis of unsaturated fatty acids | 21 | 3 | 0.000196 | 0.03787 | SCD5,ELOVL5,ELOVL2 |
| 2 | Taurine and hypotaurine metabolism | 10 | 2 | 0.000318 | 0.03787 | GAD1,CSAD |
| 3 | Tight junction | 130 | 6 | 0.002488 | 0.162812 | SPTAN1,CRB3,SYMPK,CTTN,PTEN,PARD3 |
| 4 | RNA degradation | 71 | 4 | 0.003351 | 0.162812 | TTC37,CNOT4,ZCCHC7,WDR61 |
| 5 | Insulin signaling pathway | 138 | 6 | 0.00348 | 0.162812 | GSK3B,G6PC2,PHKB,PRKAA1,RHOQ,RPTOR |
| 6 | Fatty acid elongation | 23 | 2 | 0.004105 | 0.162812 | ELOVL5,ELOVL2 |
| 7 | mTOR signaling pathway | 52 | 3 | 0.006334 | 0.184509 | PGF,PRKAA1,RPTOR |
| 8 | Endocytosis | 199 | 7 | 0.007407 | 0.184509 | CLTC,MDM2,ZFYVE16,EEA1,PIP5K1A,RAB22A,PARD3 |
| 9 | beta-Alanine metabolism | 29 | 2 | 0.007961 | 0.184509 | GAD1,ACADM |
| 10 | Hedgehog signaling pathway | 56 | 3 | 0.008229 | 0.184509 | GSK3B,CSNK1G1,CSNK1A1 |
